# Autophagy Regulates Fungal Virulence and Sexual Reproduction in *Cryptococcus neoformans*

**DOI:** 10.3389/fcell.2020.00374

**Published:** 2020-05-25

**Authors:** Su-Ting Jiang, An-Ni Chang, Lian-Tao Han, Jie-Shu Guo, Yuan-Hong Li, Tong-Bao Liu

**Affiliations:** ^1^State Key Laboratory of Silkworm Genome Biology, Southwest University, Chongqing, China; ^2^Chongqing Key Laboratory of Microsporidia Infection and Control, Southwest University, Chongqing, China

**Keywords:** *Cryptococcus neoformans*, autophagy, *ATG*s, virulence, sexual reproduction

## Abstract

Autophagy (macroautophagy) is an evolutionarily conserved degradation pathway involved in bulk degradation of cytoplasmic organelles, old protein, and other macromolecules and nutrient recycling during starvation. Extensive studies on functions of autophagy-related genes have revealed that autophagy plays a role in cell differentiation and pathogenesis of pathogenic fungi. In this study, we identified and characterized 14 core autophagy machinery genes (*ATG*s) in *C. neoformans*. To understand the function of autophagy in virulence and fungal development in *C. neoforman*s, we knocked out the 14 *ATG*s in both α and **a** mating type strain backgrounds in *C. neoformans*, respectively, by using biolistic transformation and *in vivo* homologous recombination. Fungal virulence assay showed that virulence of each *atg*Δ mutants was attenuated in a murine inhalation systemic-infection model, although virulence factor production was not dramatically impaired *in vitro*. Fungal mating assays showed that all the 14 *ATG*s are essential for fungal sexual reproduction as basidiospore production was blocked in bilateral mating between each *atg*Δ mutants. Fungal nuclei development assay showed that nuclei in the bilateral mating of each *atg*Δ mutants failed to undergo meiosis after fusion, indicating autophagy is essential for regulating meiosis during mating. Overall, our study showed that autophagy is essential for fungal virulence and sexual reproduction in *C. neoformans*, which likely represents a conserved novel virulence and sexual reproduction control mechanism that involves the autophagy-mediated proteolysis pathway.

## Introduction

*Cryptococcus neoformans* is ubiquitous encapsulated yeast pathogen causing life-threating meningoencephalitis predominantly in the immunocompromised population (Casadevall and Perfect, 1998). In recent decades, with the increase of the people having HIV/AIDS or who have received organ transplants and immunosuppressive therapy (Brown et al., 2012), *C. neoformans* infects more than one million people worldwide annually, and leading to in hundreds of thousands of people deaths per year (Park et al., 2009; Rajasingham et al., 2017). Being a human fungal pathogen, *C. neoformans* expresses several virulence factors, including the production of polysaccharide capsule and melanin, and growth at 37°C, which favors the infection and the pathogenesis of *C. neoformans* (Kozel, 1995; Kronstad et al., 2008; Zaragoza, 2019). As a heterothallic basidiomycetous fungus, *C. neoformans* has two mating types, α and **a**, and can undergo a dimorphic transition to a filamentous growth by mating and monokaryotic fruiting. During mating in *C. neoformans*, haploid cells of α and **a** mating types fused to form dikaryotic hyphae leading to the formation of a basidium, and four chains of haploid basidiospores were eventually produced on top of the basidium following the completion of meiosis inside the basidium (Figure 9; Lin and Heitman, 2006; Zhao et al., 2019b). Cryptococcal cells of a single mating type, e.g., α, can also fuse and undergo monokaryotic fruiting to produce filaments and basidiospores under laboratory conditions (Zhao et al., 2019b).

Sexual reproduction favors fungal virulence in *C. neoformans* via producing infectious spores, in that α isolates can be more virulent than congenic **a** isolates (Nielsen et al., 2005). Spores and desiccated yeast cells are thought to be the initial infectious propagules to cause infection by *Cryptococcus* as they are small enough to fit down into the deep alveoli of the lung (Velagapudi et al., 2009). Sexual reproduction also enables the pathogenic fungi to proliferate and undergo genetic exchange in response to new environmental conditions such as stressful conditions, different host organisms, or changes in the host such as antimicrobial therapy. Further research on the sexual nature of pathogenic fungi will help to elucidate how fungi have evolved into successful pathogens.

Autophagy is an evolutionally conserved cellular degradation process in which the cytoplasmic components such as organelles, aggregated proteins, invading microorganisms, and other cytoplasmic materials are sequestered and transferred to vacuole or lysosome for degradation and recycling (Yorimitsu and Klionsky, 2005). Several types of autophagy have been described, including macroautophagy (Yorimitsu and Klionsky, 2005), microautophagy (Youle and Narendra, 2011), and chaperone-mediated autophagy (Arias and Cuervo, 2011). Macroautophagy (referred to hereafter as autophagy) is the main pathway that involves the delivery of cytosolic components to the vacuole/lysosome for degradation by double-membrane vesicles known as an autophagosome. In contrast, microautophagy involves direct engulfment of cytosolic material into the vacuole/lysosome. Chaperone mediated autophagy is a complicated and specific pathway for proteolysis of specific cytosolic proteins with the aid of chaperone molecules. In all types of autophagy, the formation of the sequestering vesicles, the autophagosome, is the hallmark morphological feature of this dynamic process (Xie and Klionsky, 2007). So far, there are at least 42 autophagy-related genes (*ATG*s) that have been identified in the model yeast *S. cerevisiae* by genetic screening, and many of them are conserved in fungi, plants, and mammals (Parzych et al., 2018). Among these *ATG*s, 15 of them are required for autophagosome formation, and their corresponding gene products are referred to as the core autophagy machinery. In *S. cerevisiae*, the core autophagy machinery proteins (Atgs) are as follows: Atg1-10, Atg12, Atg13, Atg14, Atg16, and Atg18 (Xie and Klionsky, 2007).

Autophagy plays an important role in protecting the organism against diverse pathologies, including infections, cancer, neurodegeneration, aging, and heart diseases (Levine and Kroemer, 2008). The role of autophagy has also been extensively studied and appears to play a critical role in growth, morphology, development, and pathogenicity in filamentous fungi (Pollack et al., 2009; Voigt and Poggeler, 2013; Liu et al., 2016). However, of the three fungal pathogens of most significant relevance to human health, autophagy is not required for the virulence of both *Candida albicans* and *Aspergillus fumigatus*. Only *C. neoformans* has been shown to require this process during infection (Palmer et al., 2008). In *A. fumigatus*, the serine-threonine kinase *Af*Atg1 was shown to be essential for normal conidiophore development and optimal conidiation under nutrient-limiting conditions, implying that autophagy is required to recycle internal resources to support fungal development (Richie et al., 2007). However, the Af*atg1*Δ mutant remained fully virulent in a mouse model of invasive aspergillosis despite its developmental defects under starvation-associated conditions (Richie and Askew, 2008a). In *C. albicans*, an autophagy-defective mutant Ca*atg9*Δ was generated through the deletion of an *ATG9* homolog. Fungal development and virulence assay of the Ca*atg9*Δ mutant proved that autophagy is not required for *C. albicans* differentiation, survival within or killing of a macrophage cell line, and fully virulent in an intravenous mouse model of disseminated candidiasis (Palmer et al., 2007, 2008).

The role of autophagy was also investigated in *C. neoformans*, and the evidence so far suggested that autophagy also plays an important role in virulence of *C. neoformans*. For instance, disruption of the phosphatidylinositol 3-kinase (PI3K) encoding gene resulted in virulence attenuation in a murine model of cryptococcosis (Hu et al., 2008). The role of autophagy during cryptococcal infection was also confirmed by RNAi suppression of *ATG8* in the same study, which attenuated virulence in both intranasal and intravenous infection models (Hu et al., 2008). Also, the *ATG7* gene has been shown to play roles in the virulence of *C. neoformans* (Oliveira et al., 2016). Recently, four *ATG* genes, *ATG1*, *ATG7*, *ATG8*, and *ATG9*, including the two (*ATG7* and *ATG8*) studied before, were functionally examined in *C. neoformans* and the results suggested that *ATG1*, *ATG7*, *ATG8*, and *ATG9* each make different contributions to *C. neoformans* virulence, indicating *Cryptococcus* virulence may not be utterly dependent on core autophagy functions (Ding et al., 2018). More recently, Zhao et al. constructed 22 autophagy-deficient strains in *C. neoformans* and found that 12 of them showed remarkable virulence attenuation in the invertebrate (*Galleria mellonella*) model of cryptococcosis (Zhao et al., 2019a).

The above studies provided insight into the contribution of *ATG* genes to virulence to *C. neoformans*. However, only four of the 15 *ATG*s have been tested for their role in fungal virulence in the murine model of systemic cryptococcosis. The remaining 11 *ATG*s have not been studied for their role in a mouse model of cryptococcosis to our knowledge and remain to be determined. Furthermore, besides its medical importance, *C. neoformans* has been developed as a model organism for fungal genetics study because of its defined sexual cycle. However, whether the autophagy pathway regulates the sexual reproduction of *C. neoformans* and, if so, how does autophagy regulate the sexual reproduction of *C. neoformans* remains unknown.

In this study, we therefore systematically identified and characterized 14 core autophagy machinery proteins (Atgs) in *C. neoformans*. We found that each of the 14 Atgs was required for autophagy-related phenotypes. The outcome of fungal virulence study further suggested *C. neoformans* virulence may not be completely dependent on core autophagy functions as each *atg*Δ mutant displayed different levels of virulence and caused different disease progression profiles in infected mice in a murine inhalation model of cryptococcosis. Interestingly, when tested in a bilateral mating assay, all the *atg*Δ mutants failed to produce basidiospores, and the nuclei inside basidium failed to undergo meiosis after fusion, indicating Atgs are essential for regulating meiosis during mating. Overall, our study showed that autophagy is essential for fungal virulence and sexual reproduction in *C. neoformans*, which likely represents a conserved novel virulence and sexual reproduction control mechanism that involves the autophagy-mediated proteolysis pathway.

## Materials and Methods

### Strains and Growth Media

*Cryptococcus neoformans* and its derived strains used in this study are listed in Supplementary Table S1. Strains were grown at 30°C on YPD agar medium. MS medium (Murashige and Skoog medium) and V8 used for mating and sporulation assays were prepared as described previously (Xue et al., 2007). SD-N medium was used for nitrogen starvation assay (Kim et al., 2002). Minimal medium (MM) was used to induce capsule formation (Vij et al., 2018). All other media were prepared as described previously (Fan et al., 2019).

### Detection of *ATG*s Expression Under Starvation Condition

To test the expression of the *ATG*s under nitrogen starvation, we measured the *ATG*s at mRNA levels using quantitative real-time PCR (qRT-PCR) as previously described (Fan et al., 2019). Briefly, overnight cultures of the wild-type (WT) strain were washed three times with ddH_2_O and resuspended in SD-N medium for starvation induction. The harvested cells were washed with ddH_2_O, and the pellets were used for total RNA extraction and cDNA synthesis. Gene expression levels were normalized using *GAPDH* as the endogenous control gene, and the relative levels were determined using the comparative threshold cycle (C_T_) method (Livak and Schmittgen, 2001). The specificity of the PCR was further verified by subjecting the amplification products to agarose gel electrophoresis and sequencing reaction.

### Generation of *atg*Δ Mutants and Their Complemented Strains

Each of the 14 *atg*Δ mutants was generated in the WT H99 and KN99**a** strain backgrounds by using a split marker strategy with minor modification (Kim et al., 2009; Fan et al., 2019). The two overlap PCR fragments for homologous recombination were obtained after two rounds of PCR and then biolistically transformed into the cells of H99 or KN99**a** after combination and precipitation onto 10-μl of gold microcarrier beads (0.6 μm, BioRad). Stable transformants were selected on YPD plates containing 200 mg/L G418. The *ATG*s gene knockout mutants were first screened by diagnostic PCR using positive primers F4/R4 and negative primers F3/R3 (see Supplementary Table S2) and then further confirmed by Southern blotting.

To generate complemented strains of each *atg*Δ mutant, a genomic DNA fragment containing a 1.5-Kb upstream promoter region, the relevant *ATG* open reading frame (ORF), and its 500-bp downstream region was amplified in a PCR using primers Comp F1 and R1 (see Supplementary Table S2). This PCR fragment was cloned into the plasmid pTBL1, which contains the *NAT* selective marker gene to generate the complementation plasmid for each *atg*Δ mutant (see Supplementary Table S1). Each plasmid was linearized by the appropriate restriction endonuclease and biolistically transformed in both α and **a** mating-type *atg*Δ mutant strains. The mating assay was performed to identify transformants that complemented the *atg*Δ mutant phenotype.

### Nitrogen Starvation Assay

To test whether the core autophagy genes we identified in *C. neoformans* are required for autophagosome formation, the 14 *atg*Δ mutants were first cultured overnight in YPD and then washed and switched to SD-N medium supplemented with 2 mM PMSF and further incubated for 4 or 5 h. The accumulation of autophagic bodies was observed under a microscope (Olympus BX53). To examine the survival of the cryptococcal strains under nitrogen starvation conditions, the cells of WT H99, *atg*Δ mutants, and their complemented strains were first grown overnight in YPD and then washed and resuspended in SD-N medium to an OD_600_ = 1.0. At indicated times, aliquots were removed and plated onto YPD plates in triplicate after appropriate dilution. Colony-forming units of cryptococcal strains survived in nitrogen starvation were counted after 2 days of incubation at 30°C. Meanwhile, a ten-fold serial dilution of the suspensions was also prepared, and 5 μl of the aliquots were grown on YPD plates for 48 h before photography.

### Assays for Melanin, Capsule Production, and Mating

Melanin production of cryptococcal strains was performed on the Niger seed agar medium. 100 μl of each ddH_2_O washed overnight culture was grown on Niger seed plates at 30°C or 37°C for 24 or 48 h, and the pigmentation of fungal colonies was assessed. To examine capsule production, a total of 10^6^ cells from YPD overnight cultures of each strain were grown in MM medium at 30°C for 72 h. The capsule size was analyzed as described previously (Liu et al., 2011). In a mating assay, *C. neoformans* cell suspensions of opposite mating types (α or **a**) were mixed and cocultured on MS or V8 medium at 25°C in the dark. Mating filaments and basidiospore formation were examined and recorded by photography using the Olympus CX41 light microscope after incubation for 14 days.

### Virulence Studies

Overnight cultures of each yeast strains were washed twice with PBS buffer and resuspended at a final concentration of 2 × 10^6^ cells/ml. Groups of 10 female C57 BL/6 mice (Chongqing Medical University, China) were intranasally infected with 10^5^ cells of each yeast strain as previously described (Cox et al., 2000). Animals that appeared moribund or in pain were sacrificed by CO_2_ inhalation throughout the experiments. Survival data from the murine experiments were statistically analyzed between paired groups using the log-rank test, and statistical analysis of fungal burden was performed by non-parametric Mann-Whitney test with PRISM version 7.0 (GraphPad Software, San Diego, CA) (*P* values of <0.001 were considered significant).

### Histopathology and Fungal Burdens in Infected Organs

Infected animals were sacrificed at the endpoint of the experiment according to Southwest University-approved animal protocol. For mice infected by the *atg6*Δ and *atg18*Δ mutants, the experiment was terminated at 80 days post-infection (DPI). To compare the fungal burdens, brains, lungs, and spleens from mice infected by H99, each *atg*Δ mutant, or the complemented strain of each *atg*Δ mutant were isolated at the end time point (ETP), fixed in 10% formalin solution, and sent to the Servicebio biological laboratory for section preparation (Servicebio, Wuhan, China). To evaluate the disease progression, mice infected by the WT, *atg6*Δ, *atg8*Δ, and *atg12*Δ mutants were sacrificed at 7, 14, 21 DPI, and the organs were also prepared for section and fungal burden examination. Tissue slides were stained with hematoxylin and eosin (H&E) and examined by light microscopy (Olympus BX53). Infected brains, lungs, and spleens were also isolated and homogenized using a homogenizer in PBS buffer. Resuspensions were diluted, 100 μl of each dilution was spread on YPD medium with ampicillin and chloramphenicol, and colonies were determined after 2 days of incubation at 30°C.

### Ethics Statement

The animal studies conducted at Southwest University were in full compliance with “Guidelines on Ethical Treatment of Experimental Animals (2006, No. 398)” issued by the Ministry of Science and Technology of China and the “Regulation on the Management of Experimental Animals (2006, No. 195)” issued by Chongqing Municipal People’s Government. The Animal Ethics Committee of Southwest University approved all of the vertebrate studies.

## Results

### Identification of Core Autophagy Machinery Proteins (Atgs) in *C. neoformans*

To identify the orthologs of the Atgs in the H99 strain of *C. neoformans*, the 15 Atgs from *S. cerevisiae* S288c were used as quires for a BLASTp search. Meanwhile, reciprocal BLASTp was also used to ensure that the most similar sequence of each cryptococcal Atg candidate in *S. cerevisiae* was the same as that of the *S. cerevisiae* inquiry gene. Finally, we identified 14 orthologs out of the 15 *S. cerevisiae* Atgs in *C. neoformans* except for the Atg10 (Table 1).

**TABLE 1:** The core autophagy machinery proteins in *C. neoformans.*

Name	*S. cerevisae*	*C. neoformans*	Function	Identity (%)	Similarity (%)
Atg1	YGL180W	CNAG_05005	Protein serine/threonine kinase, regulates magnitude of autophagy	46	61
Atg2	YNL242W	CNAG_06732	Peripheral membrane protein, mediates the retrieval of Atg9 from the PAS back to peripheral sites	28	45
Atg3	YNR007C	CNAG_06892	E2-like conjugating enzyme, functions in the conjugation of Atg8-PE	35	48
Atg4	YNL223W	CNAG_02662	Cysteine protease, cleaves the C-terminal arginine residue from Atg8 or PE from Atg8-PE conjugate	33	47
Atg5	YPL149W	CNAG_06519	Conjugation target of Atg12, part of the Atg12-Atg5-Atg16 complex	17	33
Atg6	YPL120W	CNAG_01773	Subunit of class III PI3K complex I and II, functions in autophagy and the VPS pathway	32	47
Atg7	YHR171W	CNAG_04538	E1-like activating enzyme, functions in the conjugation of Atg12 and Atg5 or Atg8 and PE	37	52
Atg8	YBL078C	CNAG_00816	Ubiquitin-like protein, conjugated to PE, controls the phagophore expansion	78	90
Atg9	YDL149W	CNAG_01445	Transmembrane protein, cycles between the PAS and other cytosolic punctate structures	39	60
Atg12	YBR217W	CNAG_07645	Ubiquitin-like protein, conjugated to Atg5, part of the Atg12-Atg5-Atg16 complex	37	60
Atg13	YPR185W	CNAG_00778	Regulatory subunit of Atg1 complex, stimulates Atg1 activity and controls the magnitude of autophagy	36	60
Atg14	YBR128C	CNAG_03608	Autophagy-specific subunit of PI3K complex I, targets the complex I to the PAS	27	60
Atg16	YMR159C	CNAG_02576	Component of the Atg12-Atg5-Atg16 complex, mediates the oligomerization of the complex	35	62
Atg18	YFR021W	CNAG_02269	Phosphoinositide binding protein, binds PI3P and PI4P, interacts with Atg2 to mediate the retrieval of Atg9 from the PAS back to peripheral sites	35	52

**FIGURE 1:**
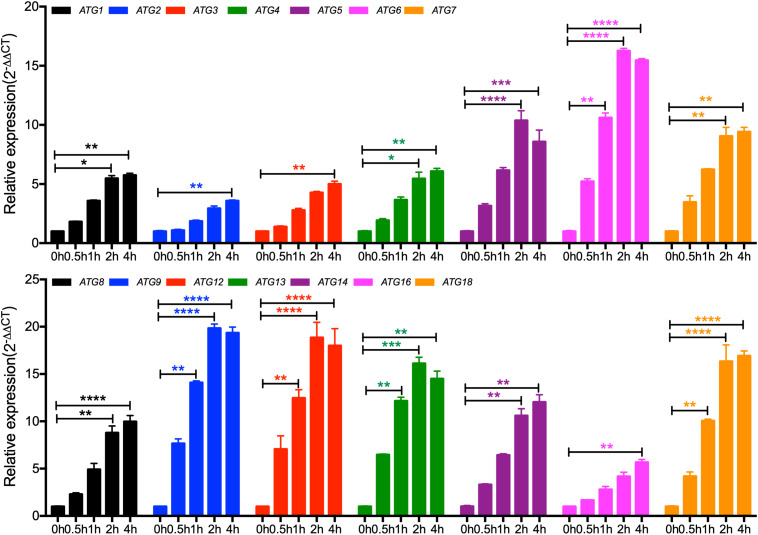
Expression of the core autophagy machinery genes (*ATG*s) under nitrogen starvation in *C. neoformans*. The expression of *ATG*s under nitrogen starvation was measured by qRT-PCR. Overnight cultures of the WT H99 strain in YPD were washed three times with ddH_2_O and resuspended in SD-N medium for the induction of nitrogen starvation. Cells were harvest after 0, 0.5, 1, 2, and 4 h induction, and RNAs were purified for cDNA synthesis and qRT-PCR analysis. The comparative *C*_T_ method was used for the relative quantification, and the *GAPDH* gene was used as an endogenous reference. Values are expressed as relative expression (2^–ΔΔCT^) of *ATG*s. Bars represent the mean ± the standard error of the mean. Statistical analysis of *ATG*s expression was done with the Kruskal-Wallis non-parametric test for multiple comparisons. *P < 0.05; **P < 0.01; ***P < 0.001; ****P < 0.0001 (for compared groups that showed statistically significant differences).

To further ensure that the identified Atg candidates in *C. neoformans* are Atgs, we detected the expression of the *ATG* candidate gene in nitrogen starvation conditions by qRT-PCR. Our results showed that the expression levels of all the 14 candidate genes increased under nitrogen starvation conditions (Figure 1), implying that the 14 candidate genes are *ATG* genes in *C. neoformans*.

### The *ATG*s Are Required for Autophagy in *C. neoformans*

**FIGURE 2:**
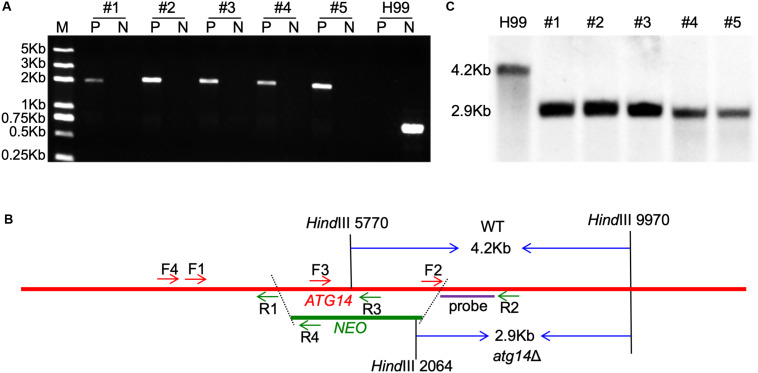
Generation of *ATG14* deletion mutants. **(A)** PCR-based verification of the G418 resistant transformants. Number 1–5 are the five G418 resistant transformants used for PCR screening; P, positive primers, TL1134/TL59 (F4/R4 in 2B); N, negative primers, TL1132/TL1133 (F3/R3 in 2B). **(B)** Restriction enzymes used for digestion of the genomic DNAs for Southern blot. The TL1130/TL1131 (F2/R2) PCR products were used as a template to synthesize the probe. The WT H99 will generate a 4.2-Kb band while the *atg14*Δ mutants generate a 2.9-Kb band. **(C)** Southern blot analysis of the *ATG14* deletion transformants. All genomic DNAs were digested with *Hin*dIII, fractionated, and hybridized with a probe located in the downstream flanking sequence of *ATG14* shown in Figure 2B. As expected, a 4.2-Kb band was detected in the WT H99 in contrast with a 2.9-Kb band in *atg14*Δ mutants.

To further investigate the roles of the *ATG* genes in *C. neoformans*, we generated the 14 single-gene deletion mutants in both H99 and KN99**a** strain backgrounds of *C. neoformans*. The 14 *ATG*s gene knockout mutants were first screened by diagnostic PCR using positive primers F4/R4 and negative primers F3/R3 (see Supplementary Table S2) and then further confirmed by Southern blotting (Figure 2 and Supplementary Figure S1). The complemented strains of each *atg*Δ mutants were also obtained by introducing the corresponding complementation plasmid in both α and **a** mating-type *atg*Δ mutants (see Supplementary Table S3 for a summary of the *ATG*-related strains).

**FIGURE 3:**
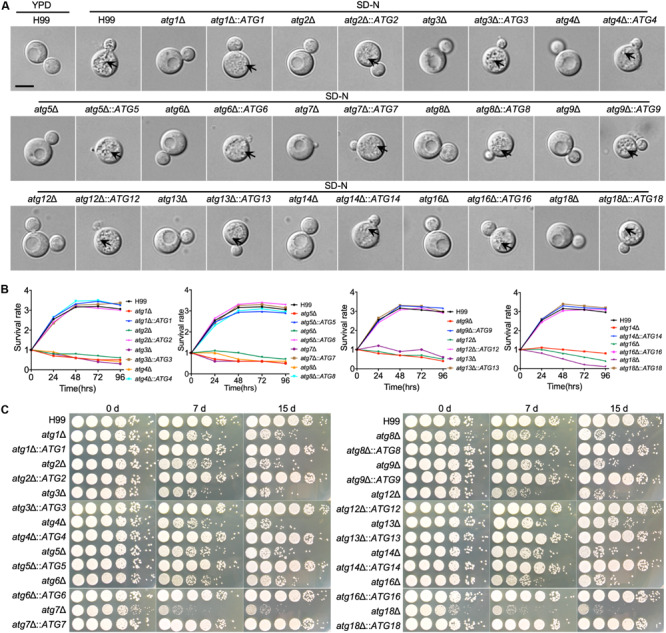
The *ATG*s are required for autophagosome formation and survival under nitrogen starvation by *C. neoformans* **(A)**. WT and *atg*Δ cells were grown on YPD medium and then switched to SD-N medium with 2 mM PMSF for nitrogen starvation induction. Cells are collected after 4 h incubation and examined under microscopy. The vacuoles of the WT strain were filled with autophagic bodies, while no autophagic body was evident in the vacuoles of the *atg*Δ mutants. The name of WT strain, *atg*Δ mutants, and their complemented strains are indicated on the top. An autophagosome is indicated by the arrow. Bars, 5 μm. **(B)** Overnight cultures of the WT, *atg*Δ mutants and their complemented strains were washed three times with ddH_2_O and diluted to an optical density at 600 nm (OD600) of 1.0 with SD-N medium. CFU was measured every 24 h and presented as relative CFU compared to that at 0 h. **(C)** Cultures of **(B)** induced in SD-N medium as indicative time were ten times diluted with ddH_2_O, and 5 μl of each were plated on YPD. The plates were incubated at 30°C for 2 days. WT H99, *atg*Δ mutants, and their complemented strains are indicated on the left and the starvation induction time on the top.

First, we verified the formation of autophagosomes of each single deletion mutant under nitrogen starvation conditions (SD-N), and the results showed that all the single deletion mutants failed to produce autophagosomes in *C. neoformans*. At the same time, more than half of the WT cells had accumulated autophagosome-like vesicles in the vacuole in the present of 2 mM PMSF (Figure 3A). The autophagosomes formation levels of the mutants were restored to the WT level when each *ATG* gene was reintroduced into the respective mutants (Figure 3A). Besides, we tested the survival of every single mutant under nitrogen starvation conditions, and each single deletion mutant exhibited the expected autophagy phenotype of impaired survival upon nitrogen starvation (Figures 3B,C). After transfer from a nutrient-rich medium, e.g., YPD to a nitrogen-free medium, e.g., SD-N, the WT cells remained viable even after 15 days of starvation induction. In contrast, cells of all the *atg*Δ mutants showed survival defects during starvation (Figure 3C). The survival rate of the *atg*Δ mutants was restored to the WT level when each *ATG* gene was reintroduced into the respective mutants (Figures 3B,C).

### Loss of Atgs Impairs Growth, Cell Membrane Integrity, and Capsule Production in *C. neoformans*

**FIGURE 4:**
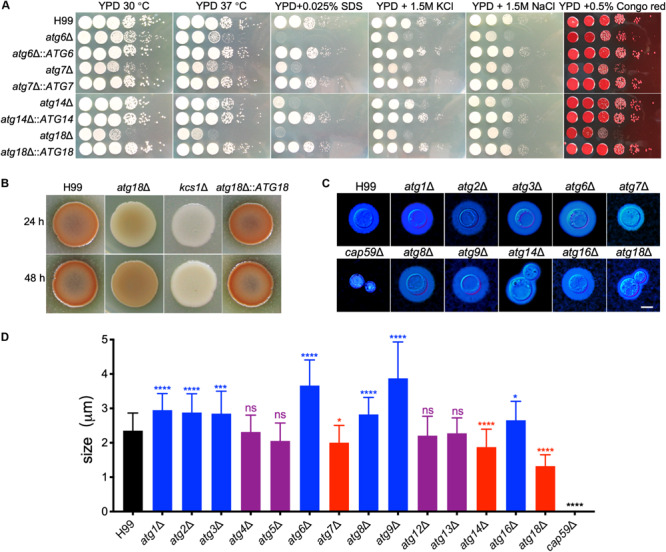
Growth and virulence factors production by cryptococcal *atg*Δ mutants. **(A)** Overnight cultures in YPD were washed with ddH_2_O three times and diluted to an optical density at 600 nm (OD600) of 2.0. Ten-fold serials were prepared in ddH_2_O, and 5 μl of each was plated on YPD or YPD with different stresses. The plates were grown for 3 days at 30°C for the inhibitor plates and the indicated temperature for all others. The conditions are indicated on the top and cryptococcal strains on the left. **(B)** Melanin production of H99 and *atg18*Δ mutants were performed in Niger seed plates. Melanin levels produced by the strains were observed in photographs after incubation for 24 and 48 h at 37°C. **(C)** Capsule formation was assayed at 30°C on MM medium. Capsule formation was visualized by India ink staining after cells grown on MM for 3 days. Bar = 5 μm. **(D)** Statistical analysis of the capsule formation in *atg*Δ mutants. Quantitative measurement of capsule size was determined by measuring the distance from the cell wall to the capsule edge (India ink exclusion zone) in 106 cells. The experiment was repeated three times. Statistical analysis of capsule sizes was done with the Kruskal-Wallis non-parametric test for multiple comparisons. ns, not significant. *P < 0.05; ***P < 0.001; ****P < 0.0001.

To investigate the roles of Atgs on cell growth and virulence in *C. neoformans*, we next examined the cell growth and the production of three major virulence factors of *atg*Δ mutants under various stress conditions. The *atg6*Δ, *atg7*Δ, and *atg18*Δ mutants had a slight growth defect on YPD plates at both 30°C and 37°C while other *atg*Δ mutants were not different from the WT strain H99 (Figure 4A). The *atg6*Δ, *atg7*Δ, *atg14*Δ, and *atg18*Δ mutants showed severe growth defects on YPD with 0.025% SDS and moderate growth defect on YPD with 1.5 M NaCl or KCl (Figure 4A), but not Congo red, indicating that Atg6, Atg7, Atg14, and Atg18 may regulate cell membrane integrity in *C. neoformans*. Additionally, *atg18*Δ mutant hardly grows on a nitrogen-deficient medium such as YNB or SD-N, implying that Atg18 is essential for nitrogen source utilization in *C. neoformans* (Supplementary Figure S2).

Besides the growth defect on YPD, the *atg18*Δ mutant produced less melanin than the WT strain after 24 or 48 h of incubation. However, the levels of melanin produced by the *atg18*Δ mutant at 48 h were comparable to those produced by the WT strain at 24 h (Figure 4B), suggesting that the less melanin production by *atg18*Δ mutant was due to impaired growth.

We next examined the capsule formation of the *atg*Δ mutants. Interestingly, we found a deletion of each *ATG* gene has different effects on cryptococcal capsule formation. The *atg4*Δ, *atg5*Δ, *atg12*Δ, and *atg13*Δ mutants were able to produce a capsule of a size similar to that of the WT strain. In contrast, deletion each of the *ATG1*, *ATG2*, *ATG3*, *ATG6*, *ATG8*, *ATG9*, and *ATG16* causes enlarged capsule, while disruption any of the *ATG7*, *ATG14*, and *ATG18* causes reduced capsule in *C. neoformans* (Figures 4C,D, *P* < 0.0001 for *ATG7* and *P* < 0.0001 for *ATG14* and *ATG18*), indicating that each of the *ATGs* has a different role in capsule formation in *C. neoformans* even though they are the Atgs.

All the defects showed in the *atg*Δ mutants were recovered when each gene was reintroduced into the respective mutants at the genomic safe haven locus (Supplementary Figure S3).

### Roles of Atgs in Fungal Infection of *C. neoformans*

**FIGURE 5:**
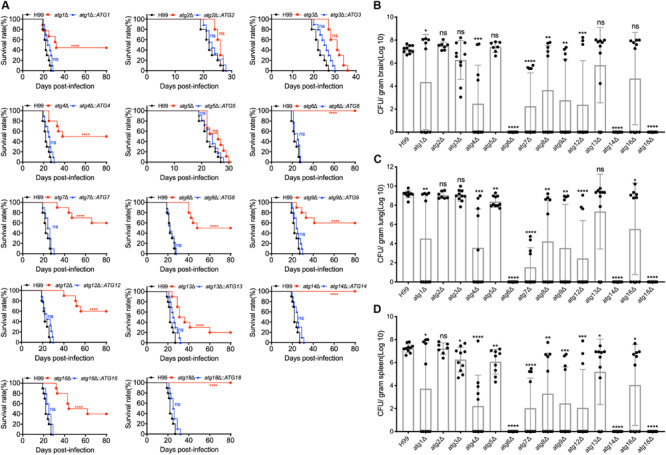
Atgs regulate virulence of *C. neoformans* in a murine inhalation model. **(A)** C57 BL/6 mice were intranasally inoculated with 10^5^ cells of H99, the 14 *atg*Δ mutants (*atg1*Δ, *atg2*Δ, *atg3*Δ, *atg4*Δ, *atg5*Δ, *atg6*Δ, *atg7*Δ, *atg8*Δ *atg9*Δ, *atg12*Δ, *atg14*Δ, *atg16*Δ, and *atg18*Δ), and their complemented strains (*atg1*Δ::*ATG1*, *atg2*Δ::*ATG2*, *atg3*Δ::*ATG3*, *atg4*Δ::*ATG4*, *atg5*Δ::*ATG5*, *atg6*Δ::*ATG6*, *atg7*Δ::*ATG7*, *atg8*Δ::*ATG8*, *atg9*Δ::*ATG9*, *atg12*Δ::*ATG12*, *atg14*Δ::*ATG14*, *atg16*Δ::*ATG16*, and *atg18*Δ::*ATG18*). The animals were monitored for clinical signs of cryptococcal infection and sacrificed at predetermined clinical endpoints that predict imminent mortality. All the *atg*Δ mutant strains except *atg2*Δ, *atg3*Δ, and *atg5*Δ are less virulent, and *atg6*Δ, *atg14*Δ, and *atg18*Δ are completely avirulent compared with the WT strain, H99. ns: not significant. ****P < 0.0001 [determined by log rank (Mantel-Cox) test]. **(B–D)** Brains, lungs, and spleens from ten mice infected with H99 and the 14 *atg*Δ mutant strains were isolated at the end time point of infection. The isolated organs were homogenized and spread on YPD plates containing ampicillin and chloramphenicol after dilution. CFU per gram of fresh organ was also measured in lung homogenates. Each data point and the error bar indicates the mean and standard error of the mean for values from five animals. *P < 0.05; **P < 0.01; ***P < 0.001; ****P < 0.0001; ns, not significant (determined by Mann-Whitney test).

To evaluate the role of Atgs in fungal virulence in *C. neoformans*, we examined the virulence of all 14 *atg*Δ mutant strains in a murine inhalation model of systemic infection. Eight-week-old female C57 BL/6 mice (*n* = 10/group) were inoculated intranasally with 10^5^ yeast cells of each *Cryptococcus* strain, and the animals were monitored twice a day. All the mice infected by the WT H99 strain died around 19 to 27 DPI. In contrast, the 14 *atg*Δ mutants showed significant differences in their ability to promote disease development in the murine inhalation model of cryptococcosis. Mice infected with the *atg2*Δ and *atg5*Δ mutant showed 100% mortality around 24 to 27 DPI and 22 to 30 DPI, respectively, with no statistical difference from the mice infected with the WT strains [*P* > 0.9999, Log-rank (Mantel-Cox) test] (Figure 5A). Mice infected by *atg3*Δ mutant survived between 27 and 36 DPI, which showed no significant difference compared with that of WT strain, even though 8 days later than WT-infected mice [*P* > 0.9999, Log-rank (Mantel-Cox) test] (Figure 5A). Meanwhile, severe organ damage with visible lesion development observed in brains, lungs, and spleens infected by *atg2*Δ, *atg3*Δ, and *atg5*Δ mutants (Supplementary Figure S4). The above results showed that loss of Atg2, Atg3, or Atg5 does not affect the virulence of *C. neoformans*. In contrast, mice infected by *atg6*Δ mutant, *atg14*Δ mutant or *atg18*Δ mutant had no illness or symptoms and continued gaining body weight until the end of the experiment (80 DPI) (Figure 5A), indicating that Atg6, Atg14, and Atg18 are essential for fungal virulence in *C. neoformans*. Fungal burden assay of the mouse organs at the endpoint of the infection showed that no yeast cells were recovered in *atg6*Δ-, *atg14*Δ-, or *atg18*Δ-infected lung, brain, and spleen (Figures 5B–D), indicating that *atg6*Δ mutant, *atg14*Δ mutant, and *atg18*Δ mutant cells were wholly cleared from the lung after inoculation.

The rest of the *atg*Δ mutants (*atg1*Δ, *atg4*Δ, *atg7*Δ, *atg8*Δ, *atg9*Δ, *atg12*Δ, *atg13*Δ, and *atg16*Δ) showed a similar ability to promote disease development in the murine model of cryptococcosis. Mice infected by the rest of the *atg*Δ mutants had a 40–80% mortality rate after 19–66 DPI, and the remaining 20–60% survived to the end of the experiment (80 dpi) (Figure 5A), which was significantly different from that of WT-infected mice [*P* < 0.0001, Log-rank (Mantel-Cox) test]. Fungal burdens in organs of the animals infected by the rest of the *atg*Δ mutants were also examined at the endpoint of the infection experiments and evaluated as yeast colony-forming unit (CFU) per gram fresh organ (See Figures 5B–D). Meanwhile, severe organ damage with visible lesion development observed in brains, lungs, and spleens of the mice died before 80 DPI (Supplementary Figure S5) while no detectable damage or lesion was detected in brains, lungs, and spleens of the mice survived at 80 DPI (Supplementary Figure S6). The complemented strains of each *atg*Δ mutant were also generated and killed the mice around 20 to 30 days after infection, confirming that the virulence attenuation phenotype in each *atg*Δ mutants is caused by the deletion of its corresponding *ATG* gene (Figure 5A).

**FIGURE 6:**
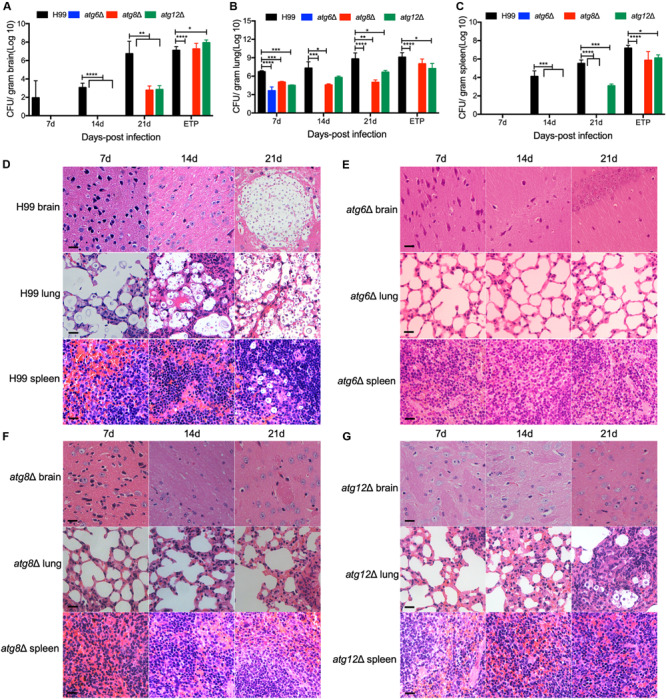
Progression of *atg*Δ mutants infection *in vivo*. Brains, lungs, and spleens from the animals infected with H99, *atg6*Δ, *atg8*Δ, and *atg12*Δ mutants were isolated at 7, 14, and 21 DPI. Colony-forming units (CFU) were measured in the brain **(A)**, lung **(B)**, and spleen **(C)** homogenates. Each data point and the error bar indicates the standard error of the mean for values from ten mice. *P < 0.05; **P < 0.01; ***P < 0.001; ****P < 0.0001 (determined by Mann-Whitney test). **(D–G)** H&E-stained slides were also prepared from brain, lung, and spleen cross-sections and visualized by light microscopy. Bars, 20 μm.

To better understand the dynamics of the *atg*Δ mutants-host interaction during the infection process, three representative mutant strains, *atg6*Δ, *atg8*Δ, and *atg12*Δ, were used to infect the mice and fungal burdens in infected brains, lungs, and spleens were examined at 7, 14, and 21 DPI and ETP. Our results showed that in *atg6*Δ mutant-infected lungs, ∼10^3^ CFU/gram fresh lung were recovered at 7 DPI and no yeast cells were recovered at 14, 21 DPI and ETP (Figure 6B), indicating that the yeast cells of *atg6*Δ mutant were gradually cleared away after inoculation. No yeast cells were recovered from the brains and spleens of the mice infected by *atg6*Δ mutant (Figures 6A,C). Fungal lesion development in the lung was also visualized in H&E-stained slides. As shown in Figure 6D, the WT strain H99 caused severe damage in infected brains, lungs, and spleens, with abundant yeast cells, as early as 7 DPI. In contrast, lungs infected by the *atg6*Δ mutant showed little damage, with very few yeast cells observed at different time points (Figure 6E). In *atg8*Δ and *atg12*Δ mutants infected mice, comparable yeast cells were recovered from brains at 21 DPI and ETP (Figure 6A), and from lungs at every time point (Figure 6B). Although no *atg8*Δ yeast cells were recovered from the spleens at 21 DPI, comparable yeast cells of *atg8*Δ mutant and *atg12*Δ mutant were recovered at the ETP (Figures 6C,F,G).

Taken together, our founding revealed that each *ATG* gene contributes differently to the virulence of *Cryptococcus* in C57 BL/6 mice. Our results also further suggest that, besides the roles of the genes in autophagy, non-autophagic functions associated with each *ATG* gene may contribute to the virulence of *Cryptococcus*.

### The Atgs Are Essential for Sexual Reproduction

**FIGURE 7:**
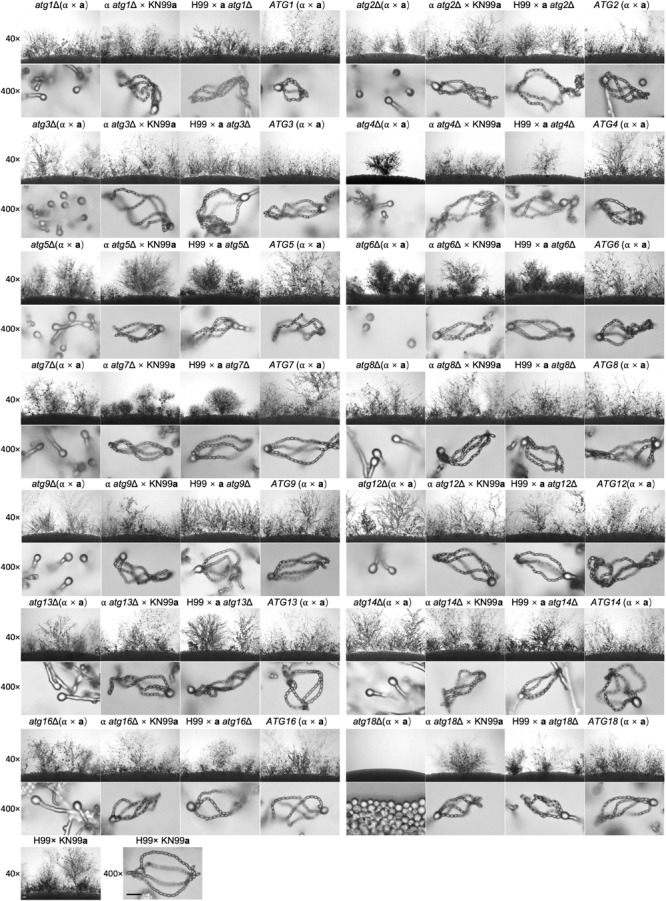
Mating filaments production and sporulation on cryptococcal *atg*Δ mutants. Bilateral matings of the WT strains (H99 × KN99**a**), *atg*Δ mutants (α × **a**), and their complemented strains (*ATGs*, α × **a**) and unilateral matings of the *atg*Δ mutants (α *atg*Δ × KN99**a**, H99 × **a** *atg*Δ) were performed on MS medium. Mating structures at × 40 magnification (top, Bar = 100 μm) and ×400 magnification (bottom, Bar = 10 μm) were photographed after 14 days of incubation in the dark at 25°C.

*Cryptococcus neoformans* is a basidiomycetous fungus having two mating types, α and **a**, and can undergo heterothallic sexual reproduction to generate dikaryotic mating hyphae and basidiospores. To evaluate the role of Atgs in fungal mating, we generated each of the 14 *atg*Δ mutants and its complemented strains in both H99 and KN99**a** strain backgrounds in *C. neoformans*, respectively. Both bilateral and unilateral matings in each *atg*Δ mutant were set up to examine the development of dikaryotic hyphae and basidiospores. Remarkably, when compared with the WT strains, all *atg*Δ mutants failed to produce basidiospores, even though they generate normal dikaryotic mating hyphae as the WT strains except *atg4*Δ and *atg18*Δ mutants in bilateral matings (Figure 7). The *atg4*Δ mutants showed a mating defect, and significant reduction of mating hyphal production and failure of basidiospore production were observed in the bilateral mating assays of *atg4*Δ mutants (Figure 7). No mating hyphae were produced by *atg18*Δ mutants in bilateral mating (Figure 7). The above results indicate that both Atg4 and Atg18 are required for α-**a** mating in *C. neoformans*. In contrast, mating hyphae and basidiospores were produced during unilateral matings in both *atg4*Δ and *atg18*Δ mutants, albeit at a slightly reduced level compared to matings between WT strains. Other *atg*Δ mutants produced normal mating hyphae and basidiospores as the WT strains in the unilateral matings (Figure 7).

Additionally, all the *atg*Δ mutants were recovered when each gene was reintroduced into the respective mutants at the genomic haven locus (Figure 7). These results clearly showed that the core autophagy machinery proteins are essential for sexual reproduction in *C. neoformans*.

### The Atgs Are Involved in Meiosis and Nuclear Division

**FIGURE 8:**
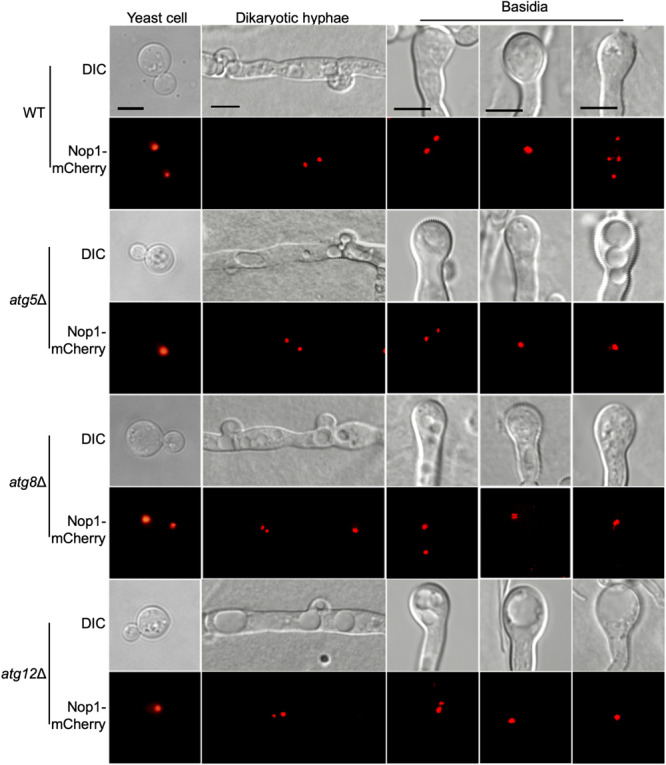
Fungal nuclei development during mating in cryptococcal *atg*Δ mutants. Fungal nuclei development assay in yeast cells, mating hyphae and basidia of the WT, *atg5*Δ mutant, *atg8*Δ mutant, and *atg12*Δ mutant strains. Mating cultures were isolated from mating plates after incubation for 7 or 14 days on MS medium in the dark and visualized under Olympus inverted confocal laser scanning microscope. Bars, 5 μm.

To investigate why *atg*Δ mutants fail to produce basidiospores, the nucleolar protein Nop1 tagged with mCherry at the C terminus (Lee and Heitman, 2012) was used to monitor the fungal nuclei development at different stages of mating in the living cells of *C. neoformans*. The *NOP1-mCherry* fusion construct was introduced into the native *NOP1* gene in both mating types of the WT and three selected *atg*Δ mutant strains (*atg5*Δ, *atg8*Δ, and *atg12*Δ) by biolistic transformation and homologous recombination (data not shown). The opposite mating strains of the WT or *atg*Δ mutants expressing Nop1-mCherry were crossed, respectively, and their nuclear positions were monitored using confocal fluorescence microscopy (Olympus, FV1200) during the mating process. A single nucleus in each yeast cell can be observed in both the WT and the *atg*Δ mutants cultures (Figure 8, first left panel), and two separated nuclei can be observed in dikaryotic hypha produced from bilateral matings after cell fusion (Figure 8, second left panel). Two separated nuclei and a single fused nucleus could be observed in the young basidium, separately, of both the WT and *atg*Δ mutants, indicating that both kinds of strains can undergo standard nuclear fusion to produce basidia during mating (Figure 8, vertical middle panel).

Interestingly, the fused nuclei in the bilateral mating of all three *atg*Δ mutants failed to undergo meiosis, and only a single nucleus could be observed in each mature basidium even after14 days of incubation, while four nuclei were produced in all basidia from WT mating (Figure 8B, right panels). The above results indicated that autophagy is critical for regulating meiosis during mating, which could help explain why all the 14 *atg*Δ mutants failed to produce spores in the bilateral mating assay. However, all the 14 *atg*Δ mutants strains have a normal growth rate and also have normal nuclear division when grown in rich medium, suggesting that autophagy may not be involved in the cell cycle during mitotic division. Overall, our results reveal that autophagy may play a role only in regulating meiosis during the mating process.

## Discussion

Autophagy is a highly conserved degradation pathway involved in bulk degradation of cytoplasmic materials for nutrient recycling during starvation, playing a critical role in cellular development and differentiation, tumor suppression, innate and adaptive immunity, and fighting against diverse pathologies (Yang and Klionsky, 2009). In this study, we systematically identified and characterized the 14 core autophagy machinery proteins required for autophagosome formation in *C. neoformans*. However, there are 15 core autophagy machinery proteins required for autophagosome formation in *S. cerevisiae*, and Atg10 is missing in *C. neoformans*. Atg10 is an E2-like conjugating enzyme and functions in the conjugation reaction between Atg12 and Atg5 in *S. cerevisiae* (Shintani et al., 1999). We used Atg10p as quires and did a BLASTp search against the *C. neoformans* H99 genome in FungiDB [(Basenko et al., 2018)] but no similar sequence was found. We also compared the sequence similarity of Atg10 and E2 conjugating enzymes in *C. neoformans* and found that the sequence similarity was very low (data not shown). Thus, there may be no Atg10 in *C. neoformans*, while other E2 conjugating enzymes perform the function of Atg10.

To investigate the role of the core autophagy machinery genes (*ATG*s), we knocked out all 14 *ATG* genes in *C. neoformans*. Each single deletion mutant showed the expected autophagy phenotype, such as impaired growth and survival upon nitrogen starvation, as previously reported by other groups (Hu et al., 2008; Oliveira et al., 2016; Gontijo et al., 2017; Ding et al., 2018; Zhao et al., 2019a; Roberto et al., 2020). However, our results suggested that autophagy-related genes may also be involved in other cellular functions, such as maintaining the integrity of cell membranes. In our study, the *atg6*Δ, *atg7*Δ, *atg14*Δ, and *atg18*Δ mutants showed severe growth defects on YPD with 0.025% SDS and moderate growth defect on YPD with 1.5 M NaCl or KCl, but not Congo red, which indicates that Atg6, Atg7, Atg14, and Atg18 may regulate cell membrane integrity in *C. neoformans* besides roles of the genes in autophagy. Other protein degradation pathways, such as the SCF E3 ubiquitin ligase-mediated ubiquitin-proteasome system (UPS), are also involved in the regulation of cell membrane integrity in *C. neoformans* (Liu et al., 2011; Liu and Xue, 2014). The UPS regulates cellular function by specifically degrading its ubiquitinated downstream target (Shabek and Ciechanover, 2010). It is now clear that autophagosomes can even recognize certain soluble proteins, such as ubiquitinated p62 and NBR1 (Kraft et al., 2010; Johansen and Lamark, 2011). Whether autophagy regulates cell membrane integrity by degrading the ubiquitinated substrates is still unknown and will be an exciting topic for future research. Meanwhile, the *atg6*Δ, *atg7*Δ, and *atg18*Δ mutants also showed slight growth defects on YPD at 30 or 37°C, which is consistent with those of other groups (Ding et al., 2018; Zhao et al., 2019a).

Additionally, our results showed that the deletion of different *ATG* genes has a different effect on the capsule formation in *C. neoformans*. The deletion of *ATG1*, *ATG2*, *ATG3*, *ATG6*, *ATG8*, *ATG9*, and *ATG16* resulted in an enlarged capsule in *C. neoformans* while the deletion of *ATG7*, *ATG14*, and *ATG18* caused the capsule to become smaller *C. neoformans* in our study. However, the previous study showed that capsule formation was not affected by the deletion of the *ATG1*, *ATG7*, *ATG8*, or *ATG9* genes (Ding et al., 2018). In our opinion, the difference in the capsule formation may be caused by the difference of the capsule-induction medium. We used the minimum medium (Vij et al., 2018) in our study while other groups used low-iron capsule-inducing medium, which can increase the capsule more than threefold (Vartivarian et al., 1993). Therefore, it is worth trying to test the difference between the two media in the effect of capsule induction in the future. In a previous study, Ding et al. (2018) showed that the *atg1*Δ, *atg8*Δ, and *atg9*Δ mutants were not different from the WT in the production of melanin, which is consistent with our results. However, in another study, Zhao et al. (2019a) showed that the deletion of *ATG6* and *ATG14* affected melanin production in *C. neoformans*, which is different from our results. The first possible reason for this discrepancy is that we used different media for melanin production; we used the Niger seed medium while other groups used the L-DOPA medium, which may lead to different results. The second possible reason is that different autophagy genes may have different roles in the melanin production in *C. neoformans*. Thus, it is also worthwhile to compare the difference between the two media in the induction of *Cryptococcus* melanin production in the future.

Moreover, the deletion of most *ATG* genes reduced the virulence of *Cryptococcus* strains in our study, although the virulence of individual *atg*Δ mutants (*atg2*Δ, *atg3*Δ, and *atg5*Δ) was not significantly different from that of WT strains. The most significant difference between our results and those of the other groups was the virulence of the *atg1*Δ mutant. Interestingly, the virulence of the *atg1*Δ mutant was reduced in our study but increased in that of the Ding et al. group (Ding et al., 2018), which may be caused by the use of different mice and different doses of inoculation for virulence study. In Ding et al. group, the female BALB/c mice were used for virulence study, and a suspension of 2 × 10^5^ cells was inoculated into each mouse. In contrast, the C57 BL/6 mice were used for virulence study, and 1 × 10^5^ cells were inoculated per mouse in our study. Another interesting thing is that the *atg4*Δ, *atg12*Δ, and *atg13*Δ mutants did show defects in fungal virulence but not involved regulation of any of the well-studied virulence factors such as capsule formation, melanin production, and growth at 37°C. This indicated that these three autophagy-related proteins might be involved in additional mechanisms or pathways of virulence regulation in *C. neoformans* except autophagy. What are mechanisms or pathways involved and whether autophagy-related proteins are related to these pathways remain to be further explored.

**FIGURE 9:**
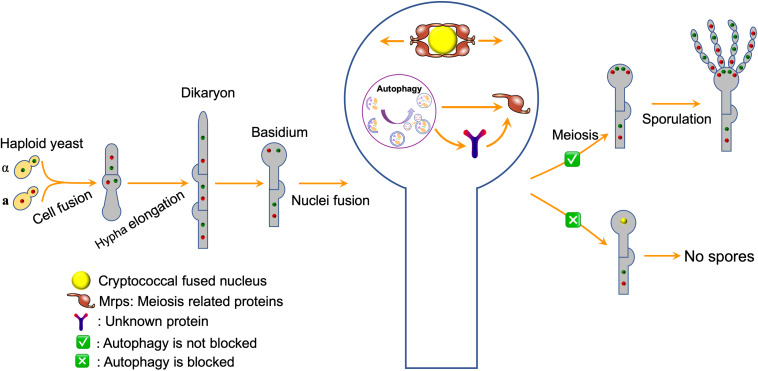
A model for autophagy regulates sexual reproduction in *C. neoformans*. The model postulates that autophagy specifically recognizes the meiosis related proteins directly or indirectly and regulates their abundance, thus regulating the sexual sporulation process of *C. neoformans*.

Previous studies showed that autophagy is required to recycle internal resources to support fungal development, such as conidiophore development and optimal conidiation in *A. fumigatus* (Richie et al., 2007; Richie and Askew, 2008b), conidiation and blastospore formation in *Beauveria bassiana* (Zhang et al., 2013; Ying et al., 2016; Chu et al., 2017), and conidiation in *Magnaporthe oryzae* (Liu et al., 2007, 2010, 2017). In the species mentioned above, blocking the autophagy pathway would affect mycelium growth and reduce conidium production, but would not result in complete loss of conidiation, indicating that autophagy is required to recycle internal resources to support fungal development. Interestingly, the deletion of the *ATG* genes blocks the basidiospore formation in *C. neoformans* but not affect the cell fusion, dikaryotic hyphae elongation, and basidium formation during the mating process except *ATG18* in our study. Fungal nuclei positioning showed that the two nuclei inside the basidium could fuse to form a nucleus but failed to undergo meiosis to form four nuclei. Based on these findings and the facts that autophagosomes can recognize certain soluble proteins, such as ubiquitinated p62 and NBR1 (Kraft et al., 2010; Johansen and Lamark, 2011), we proposed a model in which autophagy might regulate sexual reproduction of *C. neoformans* by specifically regulating meiosis related proteins (Figure 9). However, which meiosis-related protein regulates the basidiospore production of *C. neoformans*? Whether and how this meiosis-related protein is regulated by autophagy remains unknown. Thus, more data is needed to test this hypothesis.

Overall, this report described the identification and characterization of the core *ATG*s in *C. neoformans*. Our results demonstrated that the individual *ATG* genes in *C. neoformans* might contribute to virulence and sexual reproduction through participating in other cellular processes in addition to autophagy.

## Data Availability Statement

The original contributions presented in the study are included in the article/Supplementary Material, further inquiries can be directed to the corresponding author/s.

## Ethics Statement

The animal studies conducted at Southwest University were in full compliance with “Guidelines on Ethical Treatment of Experimental Animals (2006, No. 398)” issued by the Ministry of Science and Technology of China and the “Regulation on the Management of Experimental Animals (2006, No. 195)” issued by Chongqing Municipal People’s Government. The Animal Ethics Committee of Southwest University approved all of the vertebrate studies.

## Author Contributions

T-BL conceived and designed the experiments, and wrote the manuscript. T-BL, S-TJ, A-NC, and L-TH performed the experiments and acquired the data. J-SG and Y-HL contributed to the generation of cryptococcal strains. T-BL obtained the funding. All authors reviewed the manuscript and approved it for publication.

## Conflict of Interest

The authors declare that the research was conducted in the absence of any commercial or financial relationships that could be construed as a potential conflict of interest.
