# Supplementary material for: Autophagy Regulates Fungal Virulence and Sexual Reproduction in Cryptococcus neoformans
Source: Front Cell Dev Biol. 2020 May 25;8:374. doi: 10.3389/fcell.2020.00374 (PMC7262457; doi:10.3389/fcell.2020.00374)
Supplement: Supplementary file 1 [file Table_1.DOCX]

Table 1. Strains and plasmids used in this study

| Strains/plasmids | Genotype/properties | Source/reference |
| --- | --- | --- |
| *E. coli* | | |
| DH5α | cloning strain |  |
| *C. neoformans* | | |
| H99 | *MAT*α | (Perfect et al., 1993) |
| KN99**a** | *MAT***a** | (Nielsen et al., 2003) |
| TBL11 | *MAT*α *atg5*Δ::*NEO* | In this study |
| TBL12 | *MAT***a** *atg5*Δ::*NEO* | In this study |
| TBL13 | *MAT*α *atg5*Δ::*NEO ATG5*::*NAT* | In this study |
| TBL14 | *MAT***a** *atg5*Δ::*NEO ATG5*::*NAT* | In this study |
| TBL15 | *MAT*α *atg8*Δ::*NEO* | In this study |
| TBL16 | *MAT***a** *atg8*Δ::*NEO* | In this study |
| TBL17 | *MAT*α *atg8*Δ::*NEO ATG8*::*NAT* | In this study |
| TBL18 | *MAT***a** *atg8*Δ::*NEO ATG8*::*NAT* | In this study |
| TBL19 | *MAT*α *atg12*Δ::*NEO* | In this study |
| TBL20 | *MAT***a** *atg12*Δ::*NEO* | In this study |
| TBL21 | *MAT*α *atg12*Δ::*NEO ATG12*::*NAT* | In this study |
| TBL22 | *MAT***a** *atg12*Δ::*NEO ATG12*::*NAT* | In this study |
| TBL101 | *MAT*α *P_ACTIN_-NOP1-mCherry::NEO* | (Fan et al., 2019) |
| TBL102 | *MAT***a** *P_ACTIN_-NOP1-mCherry::NEO* | (Fan et al., 2019) |
| TBL116 | *MAT*α *atg8*Δ::*NEO* *P_ACTIN_-NOP1-mCherry::NAT* | In this study |
| TBL117 | *MAT***a** *atg8*Δ::*NEO* *P_ACTIN_-NOP1-mCherry::NAT* | In this study |
| TBL118 | *MAT*α *atg5*Δ::*NEO* *P_ACTIN_-NOP1-mCherry::NAT* | In this study |
| TBL119 | *MAT***a** *atg5*Δ::*NEO* *P_ACTIN_-NOP1-mCherry::NAT* | In this study |
| TBL120 | *MAT*α *atg12*Δ::*NEO* *P_ACTIN_-NOP1-mCherry::NAT* | In this study |
| TBL121 | *MAT***a** *atg12*Δ::*NEO* *P_ACTIN_-NOP1-mCherry::NAT* | In this study |
| TBL188 | *MAT*α *atg1*Δ::*NEO* | In this study |
| TBL193 | *MAT***a** *atg1*Δ::*NEO* | In this study |
| TBL194 | *MAT***a** *atg2*Δ::*NEO* | In this study |
| TBL195 | *MAT*α *atg2*Δ::*NEO* | In this study |
| TBL196 | *MAT*α *atg3*Δ::*NEO* | In this study |
| TBL197 | *MAT***a** *atg3*Δ::*NEO* | In this study |
| TBL198 | *MAT*α *atg4*Δ::*NEO* | In this study |
| TBL199 | *MAT***a** *atg4*Δ::*NEO* | In this study |
| TBL200 | *MAT*α *atg6*Δ::*NEO* | In this study |
| TBL201 | *MAT***a** *atg6*Δ::*NEO* | In this study |
| TBL202 | *MAT*α *atg9*Δ::*NEO* | In this study |
| TBL203 | *MAT***a** *atg9*Δ::*NEO* | In this study |
| TBL212 | *MAT*α *atg7*Δ::*NEO* | In this study |
| TBL213 | *MAT*a *atg7*Δ::*NEO* | In this study |
| TBL214 | *MAT*α *atg13*Δ::*NEO* | In this study |
| TBL215 | *MAT*a *atg13*Δ::*NEO* | In this study |
| TBL216 | *MAT*α *atg18*Δ::*NEO* | In this study |
| TBL217 | *MAT***a** *atg18*Δ::*NEO* | In this study |
| TBL235 | *MAT*α *atg16*Δ::*NEO* | In this study |
| TBL242 | *MAT***a** *atg16*Δ::*NEO* | In this study |
| TBL300 | *MATα atg14*Δ::*NEO* | In this study |
| TBL301 | *MATa atg14*Δ::*NEO* | In this study |
| TBL315 | *MAT*α *atg1*Δ::*NEO* *ATG1*::*NAT* | In this study |
| TBL316 | *MAT***a** *atg1*Δ::*NEO ATG1*::*NAT* | In this study |
| TBL317 | *MAT***a** *atg2*Δ::*NEO ATG2*::*NAT* | In this study |
| TBL318 | *MAT*α *atg2*Δ::*NEO ATG2*::*NAT* | In this study |
| TBL319 | *MAT*α *atg3*Δ::*NEO ATG3*::*NAT* | In this study |
| TBL320 | *MAT***a** *atg3*Δ::*NEO ATG3*::*NAT* | In this study |
| TBL321 | *MAT*α *atg4*Δ::*NEO ATG4*::*NAT* | In this study |
| TBL322 | *MAT***a** *atg4*Δ::*NEO ATG4*::*NAT* | In this study |
| TBL323 | *MAT*α *atg6*Δ::*NEO ATG6*::*NAT* | In this study |
| TBL324 | *MAT***a** *atg6*Δ::*NEO ATG6*::*NAT* | In this study |
| TBL325 | *MAT*α *atg7*Δ::*NEO ATG7*::*NAT* | In this study |
| TBL326 | *MAT*a *atg7*Δ::*NEO ATG7*::*NAT* | In this study |
| TBL327 | *MAT*α *atg9*Δ::*NEO ATG9*::*NAT* | In this study |
| TBL328 | *MAT***a** *atg9*Δ::*NEO ATG9*::*NAT* | In this study |
| TBL329 | *MAT*α *atg13*Δ::*NEO ATG13*::*NAT* | In this study |
| TBL330 | *MAT*a *atg13*Δ::*NEO ATG13*::*NAT* | In this study |
| TBL331 | *MATα atg14*Δ::*NEO ATG14*::*NAT* | In this study |
| TBL332 | *MATa atg14*Δ::*NEO ATG14*::*NAT* | In this study |
| TBL333 | *MAT*α *atg16*Δ::*NEO ATG16*::*NAT* | In this study |
| TBL334 | *MAT***a** *atg16*Δ::*NEO ATG17*::*NAT* | In this study |
| TBL335 | *MAT*α *atg18*Δ::*NEO ATG18*::*NAT* | In this study |
| TBL336 | *MAT***a** *atg18*Δ::*NEO ATG18*::*NAT* | In this study |
| Plasmids |  |  |
| pJAF1 | Ampr Plasmid harboring *NEO* marker | (Fraser et al., 2003) |
| pTBL1 | Amp^r^ Plasmid harboring *NAT* marker | (Fan et al., 2019) |
| pTBL3 | Amp^r^ Plasmid harboring *mCherry-GPD1* terminator and *NAT* marker | (Fan et al., 2019) |
| pTBL70 | Amp^r^ Vector for *P_ATG5_-ATG5-NAT* for *atg5*Δ complementation | In this study |
| pTBL72 | Ampr Vector for *P_ACTIN_-NOP1-mCherry-NAT* for nuclear positioning | (Fan et al., 2019) |
| pTBL76 | Amp^r^ Vector for *P_ATG1_*_2_*-ATG1*2*-NAT* for *atg12*Δ complementation | In this study |
| pTBL77 | Amp^r^ Vector for *P_ATG8_-ATG8-NAT* for *atg8*Δ complementation | In this study |
| pTBL189 | Amp^r^ Vector for *P_ATG3_-ATG3-NAT* for *atg3*Δ complementation | In this study |
| PTBL190 | Amp^r^ Vector for *P_ATG4_-ATG4-NAT* for *atg4*Δ complementation | In this study |
| pTBL191 | Amp^r^ Vector for *P_ATG6_-ATG6-NAT* for *atg6*Δ complementation | In this study |
| pTBL192 | Amp^r^ Vector for *P_ATG16_-ATG16-NAT* for *atg16*Δ complementation | In this study |
| pTBL193 | Amp^r^ Vector for *P_ATG18_-ATG18-NAT* for *atg18*Δ complementation | In this study |
| pTBL200 | Amp^r^ Vector for *P_ATG1_-ATG1-NAT* for *atg1*Δ complementation | In this study |
| pTBL201 | Amp^r^ Vector for *P_ATG2_-ATG2-NAT* for *atg2*Δ complementation | In this study |
| pTBL202 | Amp^r^ Vector for *P_ATG7_-ATG7-NAT* for *atg7*Δ complementation | In this study |
| pTBL203 | Amp^r^ Vector for *P_ATG9_-ATG9-NAT* for *atg9*Δ complementation | In this study |
| pTBL204 | Amp^r^ Vector for *P_ATG14_-ATG14-NAT* for *atg14*Δ complementation | In this study |

Fan, C. L., et al., 2019. The Cys2His2 zinc finger protein Zfp1 regulates sexual reproduction and virulence in *Cryptococcus neoformans*. Fungal Genet Biol. 124**,** 59-72.

Fraser, J. A., et al., 2003. Recapitulation of the sexual cycle of the primary fungal pathogen *Cryptococcus neoformans* var. *gattii*: implications for an outbreak on Vancouver Island, Canada. Eukaryot Cell. 2**,** 1036-45.

Nielsen, K., et al., 2003. Sexual cycle of *Cryptococcus neoformans* var. *grubii* and virulence of congenic a and alpha isolates. Infect Immun. 71**,** 4831-41.

Perfect, J. R., et al., 1993. Karyotyping of *Cryptococcus neoformans* as an epidemiological tool. J Clin Microbiol. 31**,** 3305-9.
