# Supplementary material for: Autophagy Regulates Fungal Virulence and Sexual Reproduction in Cryptococcus neoformans
Source: Front Cell Dev Biol. 2020 May 25;8:374. doi: 10.3389/fcell.2020.00374 (PMC7262457; doi:10.3389/fcell.2020.00374)
Supplement: Supplementary file 2 [file Table_2.DOCX]

Table S2. PCR primers used in this study

| Primers | Targeted genes | Sequence (5’-3’) |
| --- | --- | --- |
| TL17 | M13F | GTAAAACGACGGCCAG |
| TL18 | M13R | CAGGAAACAGCTATGAC |
| TL19 | *NEO* split F | GGGCGCCCGGTTCTTTTTGTCA |
| TL20 | *NEO* split R | TTGGTGGTCGAATGGGCAGGTAGC |
| TL59 | *NEO* R4 | TGTGGATGCTGGCGGAGGATA |
| TL92 | *ATG5* KO F1 | AATCCGATCGAAAGGCTCACTCACTA |
| TL93 | *ATG5* KO R1 | CTGGCCGTCGTTTTACAGGGCGGGCTGTCTGTATCCA |
| TL94 | *ATG5* KO F2 | GTCATAGCTGTTTCCTGGATGTCAAATGGGAAGGGTAGAAGGT |
| TL95 | *ATG5* KO R2 | GAAGGGCAAGGCAGATGGACAGG |
| TL96 | *ATG5* KO F3 | CCCGGTATAGGCTGAAGGATGT |
| TL97 | *ATG5* KO R3 | AACGGTCAGCGAAAAGGGTAAAGGTA |
| TL98 | *ATG5* KO F4 | GCAAAGAAGAGTAAGAGCAGAG |
| TL99 | *ATG8* KO F1 | ATGGTGGGACAGCGAGGGTGATAGTA |
| TL100 | *ATG8* KO R1 | CTGGCCGTCGTTTTACGCCTCGGCTGTGCTGGGTAATAAAG |
| TL101 | *ATG8* KO F2 | GTCATAGCTGTTTCCTGCGAGCTTCTGTGTATGATCTGTATTA |
| TL102 | *ATG8* KO R2 | GTCTTGGGTATTGCCGGTGAGTCTTT |
| TL103 | *ATG8* KO F3 | ACTGTCGGTTTGCTGTGGGTAGA |
| TL104 | *ATG8* KO R3 | GGCGCGAGCTTGATCCGTTTCCTG |
| TL105 | *ATG8* KO F4 | AACGTGAACGACTTGAGGGATGG |
| TL106 | *ATG12* KO F1 | GCGAGTTGCAAAGGTTTCATCACG |
| TL107 | *ATG12* KO R1 | CTGGCCGTCGTTTTACGAAGGCCAACCCAAATAATCCGACTG |
| TL108 | *ATG12* KO F2 | GTCATAGCTGTTTCCTGCCCGTCCACCCCTGTTAGCACC |
| TL109 | *ATG12* KO R2 | CGGTAGGCATCATCGTCTTCTCC |
| TL110 | *ATG12* KO F3 | GACGCCTCCTCCATCGGTTGTG |
| TL111 | *ATG12* KO R3 | CCCTGCGGTGGCCTTGAATA |
| TL112 | *ATG12* KO F4 | AGAATCGTAATAGCGCATGGAGGACA |
| TL115 | *ATG8* Comp F1 | GATATCGAATTCCTGCAGCCCGGGGGATCCGGTGGGGAGGAAGGAATGGGTTGA(*BamH*I) |
| TL116 | *ATG8* Comp R1 | CGGTGGCGGCCGCTCTAGAACTAGTGGATCCTTGGGTATTGCCGGTGAGTCTTT(*BamH*I) |
| TL117 | *ATG12* Comp F1 | GATATCGAATTCCTGCAGCCCGGGGGATCCATCATCTCGCGGGAAATCAGTAAC(*BamH*I) |
| TL118 | *ATG12* Comp R1 | CGGTGGCGGCCGCTCTAGAACTAGTGGATCCGGTAGGCATCATCGTCTTCTCC(*BamH*I) |
| TL119 | *ATG5* Comp F1 | GATATCGAATTCCTGCAGCCCGGGGGATCCCGGTCCTCCTCCCCTCGTTCCTTAC(*BamH*I) |
| TL120 | *ATG5* Comp R1 | CGGTGGCGGCCGCTCTAGAACTAGTGGATCCAGTTTGGGGTCGGCGGCTTGAT(*BamH*I) |
| TL217 | *GAPDH* qRT-PCR F | TGAGAAGGACCCTGCCAACA |
| TL218 | *GAPDH* qRT-PCR R | ACTCCGGCTTGTAGGCATCAA |
| TL489 | *ATG5* qRT-PCR F | CAAGAAATCGTCCCACCAA |
| TL490 | *ATG5* qRT-PCR R | CTCTGATCCAACCATCAACTC |
| TL491 | *ATG8* qRT-PCR F | GAGTGATATCCCAACGATTGAT |
| TL492 | *ATG8* qRT-PCR R | CAGAAGCATAGAGGACGTAAAG |
| TL493 | *ATG12* qRT-PCR F | AAGTTGTTGTGCGTTTCAAG |
| TL494 | *ATG12* qRT-PCR R | TCAGATGTCCTTCTGTACCA |
| TL615 | *ATG1* KO F1 | ATGTGCGCTATCGATGTATGGTG |
| TL616 | *ATG1* KO R1 | CTGGCCGTCGTTTTACGAAGATGTAAGCGGGAGTGTGAAG |
| TL617 | *ATG1* KO F2 | GTCATAGCTGTTTCCTGAAAAAGTTGGCCGAGTCATCAGGA |
| TL618 | *ATG1* KO R2 | GAAATAGGCGGGAGGAAAACTTGT |
| TL619 | *ATG1* KO F3 | AGCCCGCCACAGAGACCGAAGTAG |
| TL620 | *ATG1* KO R3 | GGGCAAATGGTGGCGAGTAGGAGA |
| TL621 | *ATG1* KO F4 | CAGCGACGAACGAACCAGCACTAT |
| TL622 | *ATG2* KO F1 | CGCTGACCGACCTTTGTTACCC |
| TL623 | *ATG2* KO R1 | CTGGCCGTCGTTTTACTCGGCCGCAGATGTCACC |
| TL624 | *ATG2* KO F2 | GTCATAGCTGTTTCCTGTAGGGGCCTCTCAAAGCTGTGGTTCG |
| TL625 | *ATG2* KO R2 | TCCCCTTGCTTCTTTTCGTTTTACTA |
| TL626 | *ATG2* KO F3 | GTTCGCTGCCCTGCTCCTCCAC |
| TL627 | *ATG2* KO R3 | GCGCGAAGTCAGAATGCACCGTAAAA |
| TL628 | *ATG2* KO F4 | CAGCGAGCGCATTGATTTGTAA |
| TL629 | *ATG3* KO F1 | TGCCGCCAAAAGAGAATGTAGGA |
| TL630 | *ATG3* KO R1 | CTGGCCGTCGTTTTACGCGTAGATGGCTGGTAGATGGAGA |
| TL631 | *ATG3* KO F2 | GTCATAGCTGTTTCCTGGCGGAAGTCGAGTTGTTTGCTGTT |
| TL632 | *ATG3* KO R2 | GGACGCTGCGGTAGTTGTGGTATT |
| TL633 | *ATG3* KO F3 | CCCACCCCATCGCCCTACAG |
| TL634 | *ATG3* KO R3 | GCACGCTCCCACCAGTCACCT |
| TL635 | *ATG3* KO F4 | ATGGCGTTGAGCGGTGGTAGATTT |
| TL636 | *ATG4* KO F1 | GGCAATAGGATCCGAGGTAAGTTC |
| TL637 | *ATG4* KO R1 | CTGGCCGTCGTTTTACGAGGTGGGGGAGGCAAGTTCGT |
| TL638 | *ATG4* KO F2 | GTCATAGCTGTTTCCTGTAGATTTGGACGAGCTTTTATGA |
| TL639 | *ATG4* KO R2 | CGCTTGACAATTTCGTTCGGTTCC |
| TL640 | *ATG4* KO F3 | CCCCGCCCTCCTCCAACTACG |
| TL641 | *ATG4* KO R3 | TCGCCGCCCCTTTTCTTTTTACTA |
| TL642 | *ATG4* KO F4 | GCTACAGCAGGGGAAACGAGGTG |
| TL643 | *ATG6* KO F1 | GAGCAGATGGAACGGGAGCAGACG |
| TL644 | *ATG6* KO R1 | CTGGCCGTCGTTTTACCGGCGGCAGATCGGAGAGTTTATG |
| TL645 | *ATG6* KO F2 | GTCATAGCTGTTTCCTGCCACTCATGCAATTTCACTTCACA |
| TL646 | *ATG6* KO R2 | GCCGAGCGCTCTGCCCTTCTA |
| TL647 | *ATG6* KO F3 | AGATTCCGCCCTGCACCCTAAA |
| TL648 | *ATG6* KO R3 | CTAAGAGTTGCCGCAGACATTCA |
| TL649 | *ATG6* KO F4 | GACTCAGACTCAGACGGGGACACC |
| TL650 | *ATG7* KO F1 | GTCGAAATGTGGGGCGGGTGAGAG |
| TL651 | *ATG7* KO R1 | CTGGCCGTCGTTTTACAGATGATGCGTAGAGGCGAGGTTA |
| TL652 | *ATG7* KO F2 | GTCATAGCTGTTTCCTGAGTCCGTCGCCGCTTACAC |
| TL653 | *ATG7* KO R2 | TCTCATGCATCCACACCGTCCTCT |
| TL654 | *ATG7* KO F3 | CTCTCCCCGCTTCCCCTTCAA |
| TL655 | *ATG7* KO R3 | GATCAAACGCCTCGCCTTCTACA |
| TL656 | *ATG7* KO F4 | CGAGGATGAGCCCGCCCAACAGAC |
| TL657 | *ATG9* KO F1 | TGGCTGTCAAGGGGAAGAGATACC |
| TL658 | *ATG9* KO R1 | CTGGCCGTCGTTTTACAGACGTGGAAGAAGAGCCGAAGTG |
| TL659 | *ATG9* KO F2 | GTCATAGCTGTTTCCTGAACACAGAGCAGCGGGAAGAATAA |
| TL660 | *ATG9* KO R2 | AGATAAAGTCGGATGCCTGCTCAA |
| TL661 | *ATG9* KO F3 | CCCGCTAAGTCGCCCAGGAT |
| TL662 | *ATG9* KO R3 | TCGCGCCAAAGCTTAGGAACT |
| TL663 | *ATG9* KO F4 | TGACGCAGCAGTTGAAAGAATGTG |
| TL664 | *ATG13* KO F1 | CAACAACGGCGTGGGGGTAAGGT |
| TL665 | *ATG13* KO R1 | CTGGCCGTCGTTTTACAGGTGGCCAGGGGAGTGATGTGC |
| TL666 | *ATG13* KO F2 | GTCATAGCTGTTTCCTGGAAGGCCCCGGGAATACCAGAAA |
| TL667 | *ATG13* KO R2 | AAAGATCCAAGGCAAGGCGAAATA |
| TL668 | *ATG13* KO F3 | CCTCGGCCGGCTCATTCTCTA |
| TL669 | *ATG13* KO R3 | CTCCCCTAAACTACCTGCCATACG |
| TL670 | *ATG13* KO F4 | ACATCCATCGAGTCGCTTAGAATC |
| TL678 | *ATG16* KO F1 | CACGTTATCGCTGGCCTATCTTTG |
| TL679 | *ATG16* KO R1 | CTGGCCGTCGTTTTACAGGCGCTCGCGAATGACGGACTG |
| TL680 | *ATG16* KO F2 | GTCATAGCTGTTTCCTGAAGAAGGGGAGGAAGGATGGGAAATA |
| TL681 | *ATG16* KO R2 | TCGTTGACGGGGGCTGAGTG |
| TL682 | *ATG16* KO F3 | GGAGGTGGTGGTGGTGGTGGTGGAC |
| TL683 | *ATG16* KO R3 | CAGTGCCCGCGGGAGGTGTTGG |
| TL684 | *ATG16* KO F4 | ACCTGGCCACATTGTACATTGATTA |
| TL685 | *ATG18* KO F1 | TGTCGGTGAGTGAGCTGTGTAATGAG |
| TL686 | *ATG18* KO R1 | CTGGCCGTCGTTTTACGAGAGGGAAGCGGGGGAGACTGAC |
| TL687 | *ATG18* KO F2 | GTCATAGCTGTTTCCTGCACCGCCGAGCTCGTAGTATGTC |
| TL688 | *ATG18* KO R2 | CAGTATGGGTGGTATGCAGAGTTTGA |
| TL689 | *ATG18* KO F3 | ATAGCGCCAATCAACCATCTGTG |
| TL690 | *ATG18* KO R3 | AATCTTCGCTGCCCAATAACCTGT |
| TL691 | *ATG18* KO F4 | GACAGCAATCGAAGAAGGAGCAGA |
| TL699 | *ATG1* qRT-PCR F1 | TGATACCCTTCTCTTCCCAGA |
| TL700 | *ATG1* qRT-PCR R1 | GATAGGCACCATGAGTCTTTCA |
| TL701 | *ATG2* qRT-PCR F1 | TTTCTGGTGTTTCTCCCATCC |
| TL702 | *ATG2* qRT-PCR R1 | CTTCGGCTCGCTCTAGAATTAC |
| TL703 | *ATG3* qRT-PCR F1 | TGGGAAGGAAGAGAAGGAGAA |
| TL704 | *ATG3* qRT-PCR R1 | GAGGGCAGTGGACGTAGTA |
| TL705 | *ATG4* qRT-PCR F1 | TCCATAGGGTCGATCTCTCTTC |
| TL706 | *ATG4* qRT-PCR R1 | AGCTCTCCATCCTGTTCCT |
| TL707 | *ATG6* qRT-PCR F1 | CATTCGCTTGCCGAGTATTTC |
| TL708 | *ATG6* qRT-PCR R1 | GCGATCTGTTTCACTCTCCA |
| TL709 | *ATG7* qRT-PCR F1 | GGGACAGATGTATCAGTGGAAA |
| TL710 | *ATG7* qRT-PCR R1 | CTCGCTATCCCAATCCAACC |
| TL711 | *ATG9* qRT-PCR F1 | TCACTTGCATCGACCTGATATT |
| TL712 | *ATG9* qRT-PCR R1 | TAAACCCACCACACCTTCATC |
| TL713 | *ATG13* qRT-PCR F1 | TTGGGCCCATTGGAAGATAC |
| TL714 | *ATG13* qRT-PCR R1 | CGCGTCTCTCAATCCCTAATC |
| TL715 | *ATG14* qRT-PCR F1 | TCAGCGAAATGATAGTGTGAGG |
| TL716 | *ATG14* qRT-PCR R1 | CACTGTCCGTACTACCCAAATC |
| TL717 | *ATG16* qRT-PCR F1 | GAGGGATAAGATACGTGCGATG |
| TL718 | *ATG16* qRT-PCR R1 | CCTTCTTCTGCTCTTCCTGAAT |
| TL719 | *ATG18* qRT-PCR F1 | ACCTCAGAGAGATTTCGCATTT |
| TL720 | *ATG18* qRT-PCR R1 | ATCCCGTTGGATCCATTTCC |
| TL1128 | *ATG14* KO F1 | ATTTGTTTGCGGTCTGGGCTGAGT |
| TL1129 | *ATG14* KO R1 | CTGGCCGTCGTTTTACCACCTGTCCGTAACCACCGATTCC |
| TL1130 | *ATG14* KO F2 | GTCATAGCTGTTTCCTGGCGCCGGCTGGACGAAAAAT |
| TL1131 | *ATG14* KO R2 | CAGCGGCCCCTATAGACGATGGTA |
| TL1132 | *ATG14* KO F3 | GCATATCTCCGCCCAACGCTAACA |
| TL1133 | *ATG14* KO R3 | GGGAGGGGGCAAAGGGGCTACAT |
| TL1134 | *ATG14* KO F4 | AGGACCTGCTTGAAATCTACACG |
| TL1135 | *ATG1* Comp F1 | GATATCGAATTCCTGCAGCCCGGGGGATCCATGTGCGCTATCGATGTATGGTG(*BamH*I) |
| TL1136 | *ATG1* Comp R1 | CGGTGGCGGCCGCTCTAGAACTAGTGGATCCGAAATAGGCGGGAGGAAAACTTGT(*BamH*I) |
| TL1137 | *ATG2* Comp F1 | GATATCGAATTCCTGCAGCCCGGGGGATCCCGCTGACCGACCTTTGTTACCC(*BamH*I) |
| TL1138 | *ATG2* Comp R1 | CGGTGGCGGCCGCTCTAGAACTAGTGGATCCTCCCCTTGCTTCTTTTCGTTTTACTA(*BamH*I) |
| TL1139 | *ATG3* Comp F1 | GATATCGAATTCCTGCAGCCCGGGGGATCCTGCCGCCAAAAGAGAATGTAGGA(*BamH*I) |
| TL1140 | *ATG3* Comp R1 | CGGTGGCGGCCGCTCTAGAACTAGTGGATCCGGACGCTGCGGTAGTTGTGGTATT(*BamH*I) |
| TL1141 | *ATG4* Comp F1 | GATATCGAATTCCTGCAGCCCGGGGGATCCGGCAATAGGATCCGAGGTAAGTTC(*BamH*I) |
| TL1142 | *ATG4* Comp R1 | CGGTGGCGGCCGCTCTAGAACTAGTGGATCCCGCTTGACAATTTCGTTCGGTTCC(*BamH*I) |
| TL1143 | *ATG6* Comp F1 | GATATCGAATTCCTGCAGCCCGGGGGATCCGACTCAGACTCAGACGGGGACACC(*BamH*I) |
| TL1144 | *ATG6* Comp R1 | CGGTGGCGGCCGCTCTAGAACTAGTGGATCCGCCGAGCGCTCTGCCCTTCTA(*BamH*I) |
| TL1145 | *ATG7* Comp F1 | GATATCGAATTCCTGCAGCCCGGGGGATCCCGAGGATGAGCCCGCCCAACAGAC(*BamH*I) |
| TL1146 | *ATG7* Comp R1 | CGGTGGCGGCCGCTCTAGAACTAGTGGATCCTCTCATGCATCCACACCGTCCTCT(*BamH*I) |
| TL1147 | *ATG9* Comp F1 | GATATCGAATTCCTGCAGCCCGGGGGATCCTGGCTGTCAAGGGGAAGAGATACC(*BamH*I) |
| TL1148 | *ATG9* Comp R1 | CGGTGGCGGCCGCTCTAGAACTAGTGGATCCAGATAAAGTCGGATGCCTGCTCAA(*BamH*I) |
| TL1149 | *ATG13* Comp F1 | GATATCGAATTCCTGCAGCCCGGGGGATCCCAACAACGGCGTGGGGGTAAGGT(*BamH*I) |
| TL1150 | *ATG13* Comp R1 | CGGTGGCGGCCGCTCTAGAACTAGTGGATCCAAAGATCCAAGGCAAGGCGAAATA(*BamH*I) |
| TL1151 | *ATG14* Comp F1 | GATATCGAATTCCTGCAGCCCGGGGGATCCATTTGTTTGCGGTCTGGGCTGAGT(*BamH*I) |
| TL1152 | *ATG14* Comp R1 | CGGTGGCGGCCGCTCTAGAACTAGTGGATCCAGCGGCCCCTATAGACGATGGTA(*BamH*I) |
| TL1153 | *ATG16* Comp F1 | GATATCGAATTCCTGCAGCCCGGGGGATCCCACGTTATCGCTGGCCTATCTTTG(*BamH*I) |
| TL1154 | *ATG16* Comp R1 | CGGTGGCGGCCGCTCTAGAACTAGTGGATCCTCGTTGACGGGGGCTGAGTG(*BamH*I) |
| TL1155 | *ATG18* Comp F1 | GATATCGAATTCCTGCAGCCCGGGGGATCCGACAGCAATCGAAGAAGGAGCAGA(*BamH*I) |
| TL1156 | *ATG18* Comp R1 | CGGTGGCGGCCGCTCTAGAACTAGTGGATCCCAGTATGGGTGGTATGCAGAGTTTGA(*BamH*I) |
