# Supplementary material for: Autophagy Regulates Fungal Virulence and Sexual Reproduction in Cryptococcus neoformans
Source: Front Cell Dev Biol. 2020 May 25;8:374. doi: 10.3389/fcell.2020.00374 (PMC7262457; doi:10.3389/fcell.2020.00374)
Supplement: Supplementary file 3 [file Table_3.DOCX]

| *ATGs* | Gene ID | *atg*Δ (⍺) | No. of isolates | *atg*Δ (**a**) | No. of isolates | Complemented strains (⍺) | No. of isolates | Complemented strains (**a**) | No. of isolates |
| --- | --- | --- | --- | --- | --- | --- | --- | --- | --- |
| *ATG1* | CNAG_05005 | *MAT*⍺ *atg1*Δ | 4 | *MAT***a** *atg1*Δ | 3 | *MAT*⍺ *atg1*Δ::*ATG1* | 4 | *MAT***a** *atg1*Δ::*ATG1* | 3 |
| *ATG2* | CNAG_06732 | *MAT*⍺ *atg2*Δ | 4 | *MAT***a** *atg2*Δ | 5 | *MAT*⍺ *atg2*Δ::*ATG2* | 4 | *MAT***a** *atg2*Δ::*ATG2* | 4 |
| *ATG3* | CNAG_06892 | *MAT*⍺ *atg3*Δ | 5 | *MAT***a** *atg3*Δ | 5 | *MAT*⍺ *atg3*Δ::*ATG3* | 5 | *MAT***a** *atg3*Δ::*ATG3* | 4 |
| *ATG4* | CNAG_02662 | *MAT*⍺ *atg4*Δ | 5 | *MAT***a** *atg4*Δ | 4 | *MAT*⍺ *atg4*Δ::*ATG4* | 4 | *MAT***a** *atg4*Δ::*ATG4* | 5 |
| *ATG5* | CNAG_06519 | *MAT*⍺ *atg5*Δ | 3 | *MAT***a** *atg5*Δ | 4 | *MAT*⍺ *atg5*Δ::*ATG5* | 3 | *MAT***a** *atg5*Δ::*ATG5* | 4 |
| *ATG6* | CNAG_01773 | *MAT*⍺ *atg6*Δ | 6 | *MAT***a** *atg6*Δ | 5 | *MAT*⍺ *atg6*Δ::*ATG6* | 4 | *MAT***a** *atg6*Δ::*ATG6* | 4 |
| *ATG7* | CNAG_04538 | *MAT*⍺ *atg7*Δ | 5 | *MAT***a** *atg7*Δ | 5 | *MAT*⍺ *atg7*Δ::*ATG7* | 4 | *MAT***a** *atg7*Δ::*ATG7* | 4 |
| *ATG8* | CNAG_00816 | *MAT*⍺ *atg8*Δ | 8 | *MAT***a** *atg8*Δ | 4 | *MAT*⍺ *atg8*Δ::*ATG8* | 3 | *MAT***a** *atg8*Δ::*ATG8* | 3 |
| *ATG9* | CNAG_01445 | *MAT*⍺ *atg9*Δ | 5 | *MAT***a** *atg9*Δ | 3 | *MAT*⍺ *atg9*Δ::*ATG9* | 4 | *MAT***a** *atg9*Δ::*ATG9* | 4 |
| *ATG12* | CNAG_07645 | *MAT*⍺ *atg12*Δ | 3 | *MAT***a** *atg12*Δ | 4 | *MAT*⍺ *atg12*Δ::*ATG12* | 3 | *MAT***a** *atg12*Δ::*ATG12* | 4 |
| *ATG13* | CNAG_00778 | *MAT*⍺ *atg13*Δ | 5 | *MAT***a** *atg13*Δ | 3 | *MAT*⍺ *atg13*Δ::*ATG13* | 4 | *MAT***a** *atg13*Δ::*ATG13* | 5 |
| *ATG14* | CNAG_03608 | *MAT*⍺ *atg14*Δ | 5 | *MAT***a** *atg14*Δ | 5 | *MAT*⍺ *atg14*Δ::*ATG14* | 5 | *MAT***a** *atg14*Δ::*ATG14* | 4 |
| *ATG16* | CNAG_02576 | *MAT*⍺ *atg16*Δ | 5 | *MAT***a** *atg16*Δ | 7 | *MAT*⍺ *atg16*Δ::*ATG16* | 4 | *MAT***a** *atg16*Δ::*ATG16* | 3 |
| *ATG18* | CNAG_02269 | *MAT*⍺ *atg18*Δ | 6 | *MAT***a** *atg18*Δ | 3 | *MAT*⍺ *atg18*Δ::*ATG18* | 4 | *MAT***a** *atg18*Δ::*ATG18* | 4 |

Table 3. Summary of the *ATG*-related strains constructed in this study
