# Supplementary material for: Autophagy Regulates Fungal Virulence and Sexual Reproduction in Cryptococcus neoformans
Source: Front Cell Dev Biol. 2020 May 25;8:374. doi: 10.3389/fcell.2020.00374 (PMC7262457; doi:10.3389/fcell.2020.00374)
Supplement: Supplementary file 4 [file Table_4.DOCX]

**Supplemental figure legends**

**Figure S1. Verification of *atg*Δ mutants by Southern blot.**

Southern blot analysis of the remaining 13 *ATG* deletion mutants. All genomic DNAs of each *atg*Δ mutants were digested with an appropriate restriction enzyme, fractionated, and hybridized with a probe located in the upstream or downstream flanking sequence of each *ATG* gene. The WT strain and each *atg*Δ mutants produce bands of different sizes.

**Figure S2. Growth of cryptococcal *atg*Δ mutants under stress conditions.**

Overnight cultures in YPD were washed with ddH_2_O three times and diluted to an optical density at 600 nm (OD600) of 2.0. Ten-fold serials were prepared in ddH_2_O, and 5 μl of each was plated on YPD and YPD with different stresses. The plates were grown for 2 at 30 °C for the inhibitor plates and the indicated temperature for all others. The conditions are indicated on the top and cryptococcal strains on the left.

**Figure S3. Virulence factors production by cryptococcal *atg*Δ mutants.**

(A). Melanin production of H99 and *atg*Δ mutants were performed in Niger seed plates. Melanin levels produced by the strains were observed in photographs after incubation for 24 and 48 h at 37 °C. (B). Capsule formation of *atg*Δ complemented strains was assayed at 30 °C on MM medium. Capsule formation was visualized by India ink staining after cells grown on MM for 3 days. (C). Statistical analysis of the capsule formation in *atg*Δ complemented strains. Quantitative measurement of capsule size was determined by measuring the distance from the cell wall to the capsule edge (India ink exclusion zone) in 106 cells. The experiment was repeated three times.

**Figure S4. H&E-stained slides from the organs of the mice infected by WT, *atg2*Δ, *atg3*Δ, and *atg5*Δ mutants.**

Brains, lungs, and spleens from the mice infected by WT, *atg2*Δ, *atg3*Δ, and *atg5*Δ mutants were isolated at the end time point of infection. H&E-stained slides were prepared from brain, lung, and spleen cross-sections and visualized by light microscopy. Bars, 20 µm.

**Figure S5. H&E-stained slides from the organs of the mice infected by WT and *atg*Δ mutants.**

Brains, lungs, and spleens from the mice infected by WT, *atg1*Δ, *atg4*Δ, *atg7*Δ, *atg8*Δ, *atg9*Δ, *atg12*Δ, *atg13*Δ, and *atg16*Δ mutants were isolated at the end time point of infection (before 80 DPI). H&E-stained slides were prepared from brain, lung, and spleen cross-sections and visualized by light microscopy. Bars, 20 µm.

**Figure S6. H&E-stained slides from the organs of the mice infected by WT and *atg*Δ mutants.**

Brains, lungs, and spleens from the mice infected by WT, *atg1*Δ, *atg4*Δ, *atg6*Δ, *atg7*Δ, *atg8*Δ, *atg9*Δ, *atg12*Δ, *atg13*Δ, *atg14*Δ, *atg16*Δ, and *atg18*Δ mutants were isolated at the end time point of infection (80 DPI). H&E-stained slides were prepared from brain, lung, and spleen cross-sections and visualized by light microscopy. Bars, 20 µm.
