# Supplementary figures and images for: Autophagy Regulates Fungal Virulence and Sexual Reproduction in Cryptococcus neoformans
Source: Front Cell Dev Biol. 2020 May 25;8:374. doi: 10.3389/fcell.2020.00374 (PMC7262457; doi:10.3389/fcell.2020.00374)

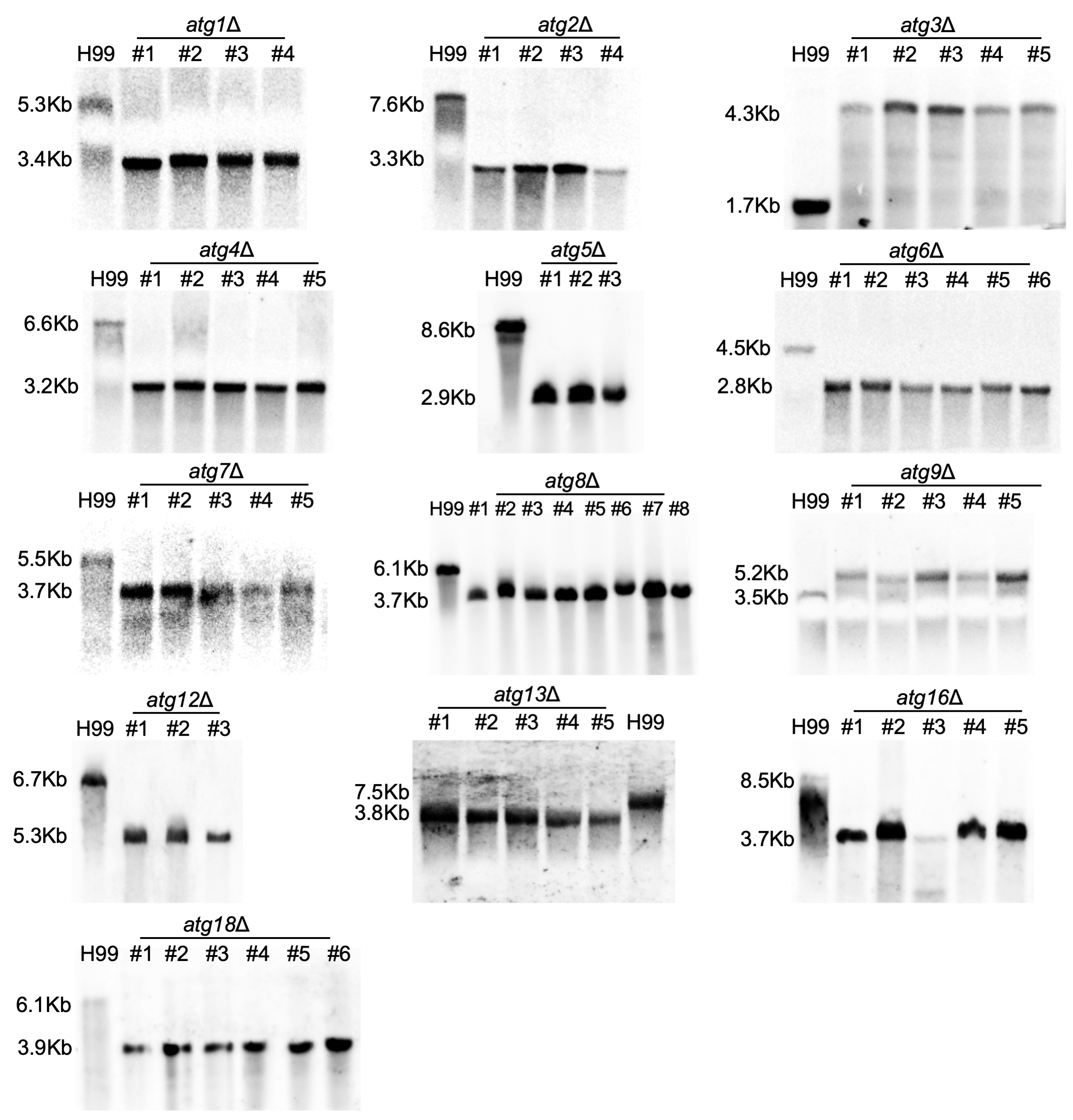

Supplement: Supplementary file 5 [file Image_1.TIF]

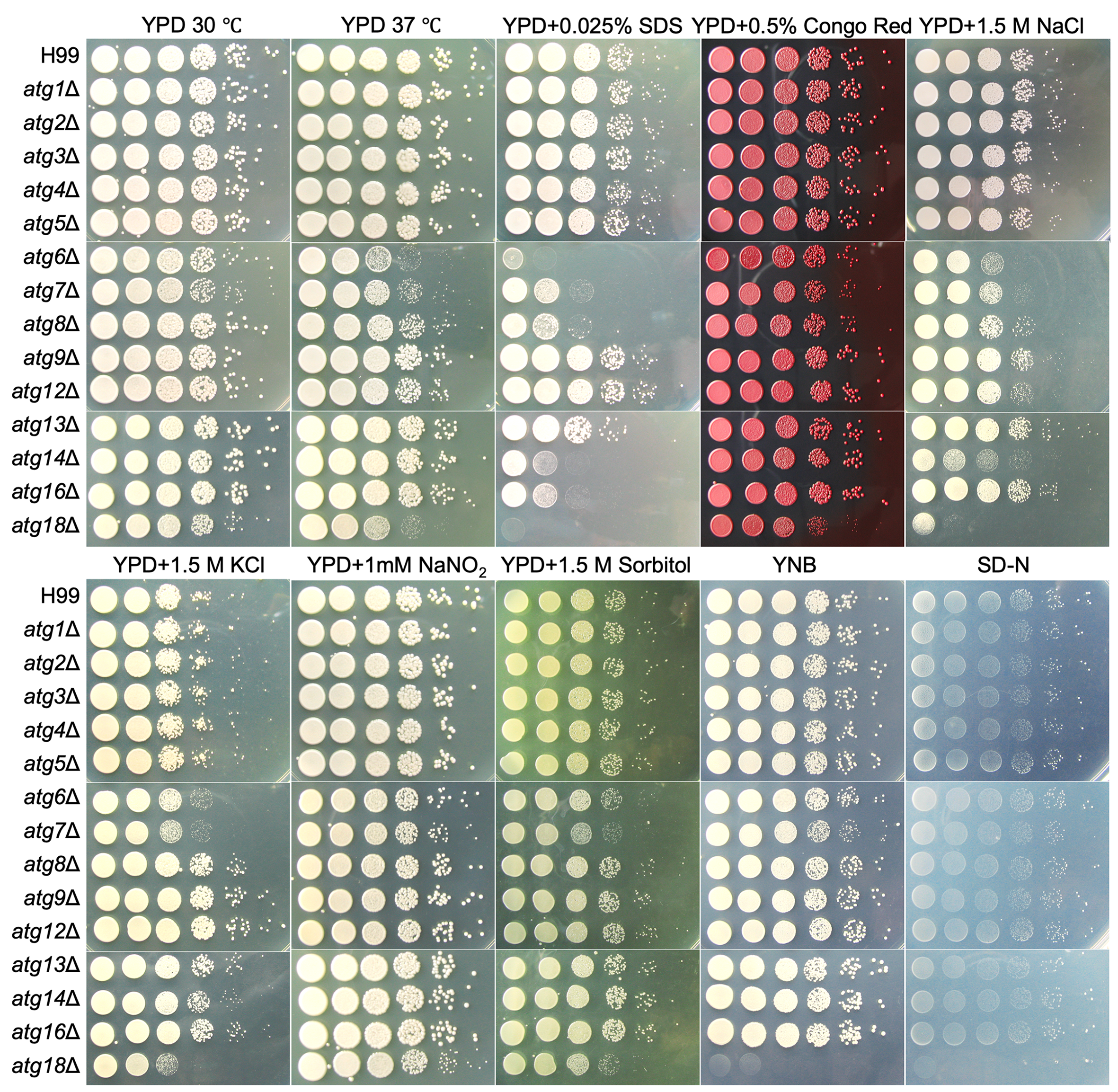

Supplement: Supplementary file 6 [file Image_2.TIF]

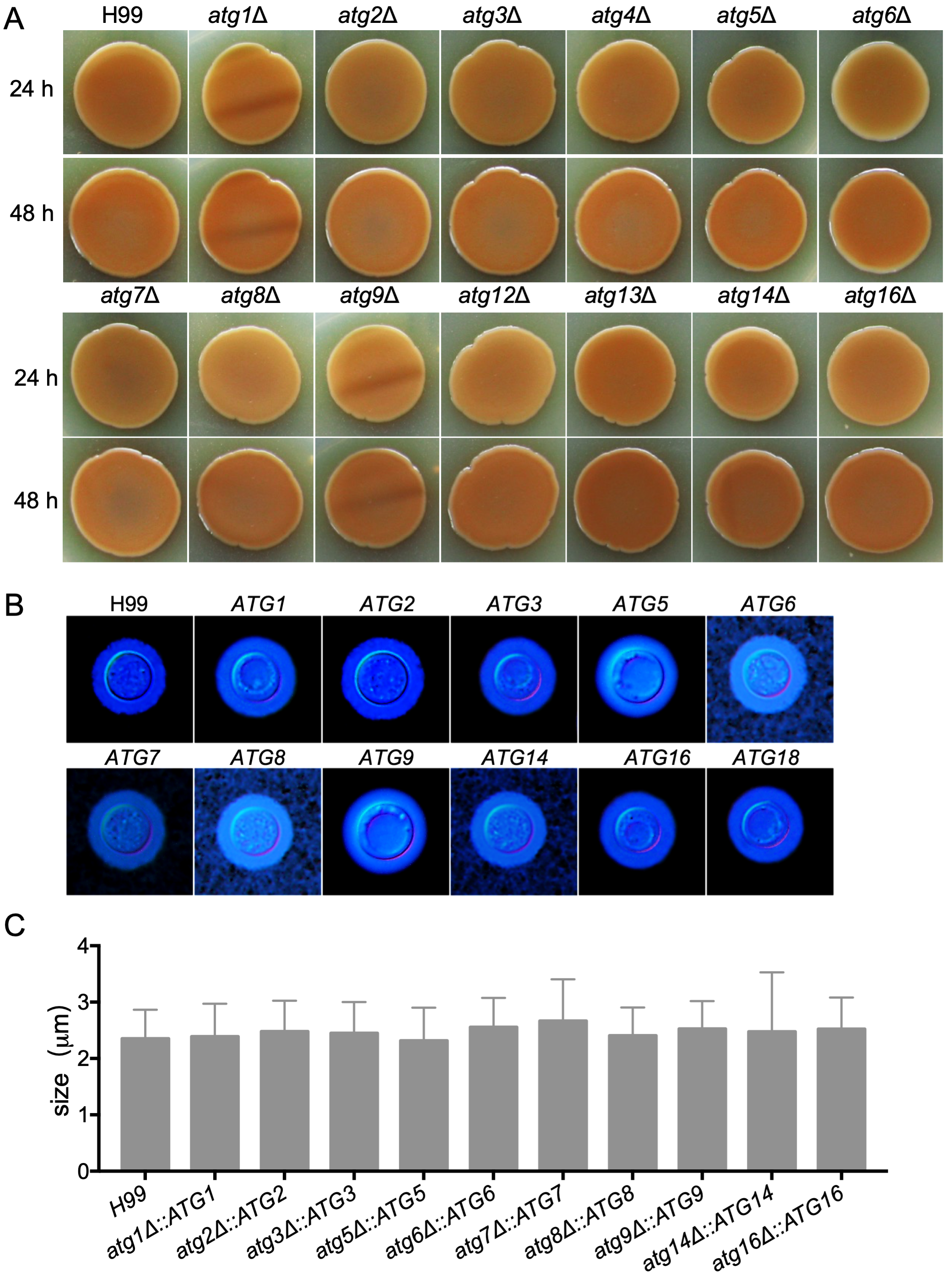

Supplement: Supplementary file 7 [file Image_3.TIF]

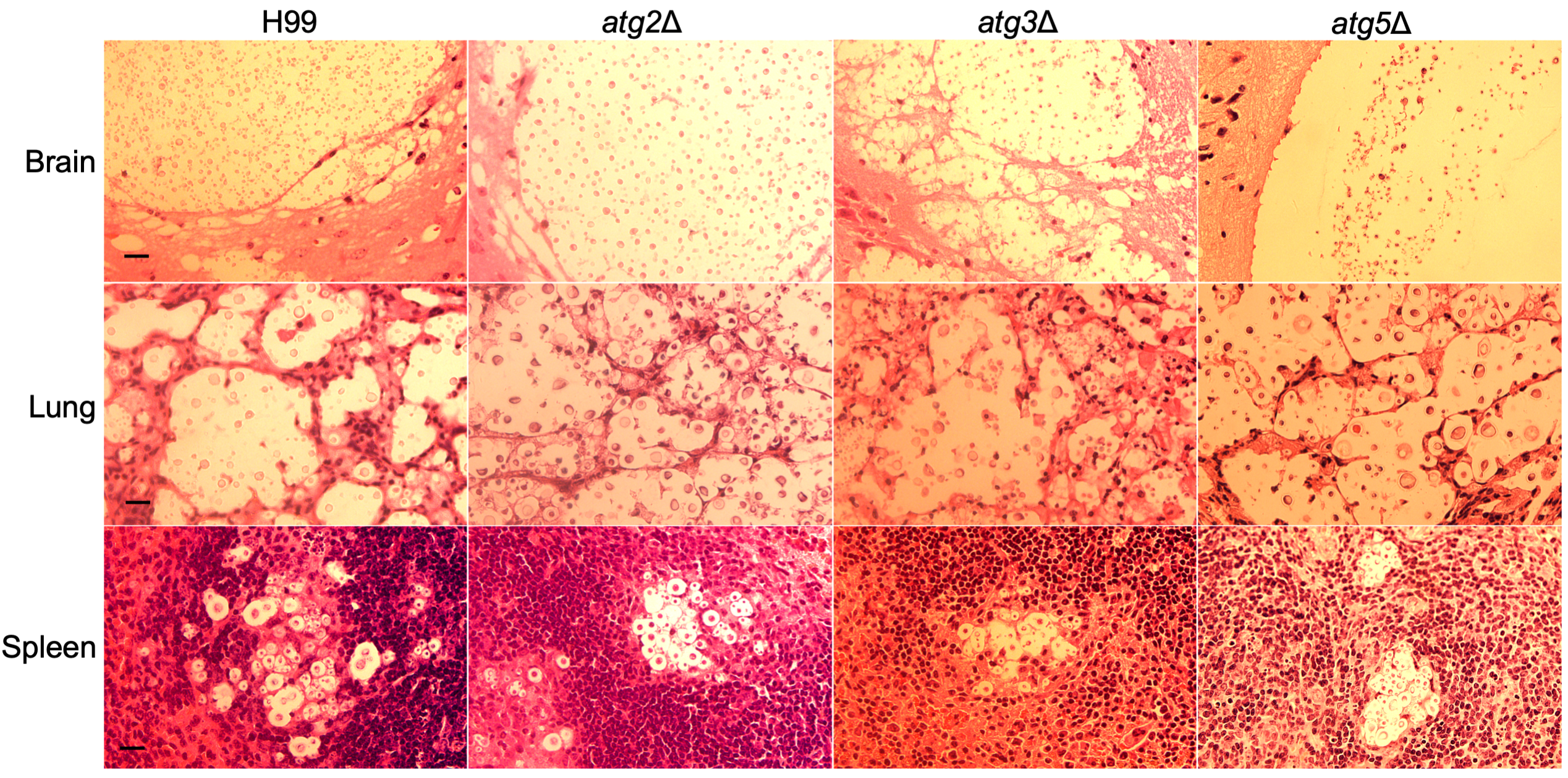

Supplement: Supplementary file 8 [file Image_4.TIF]

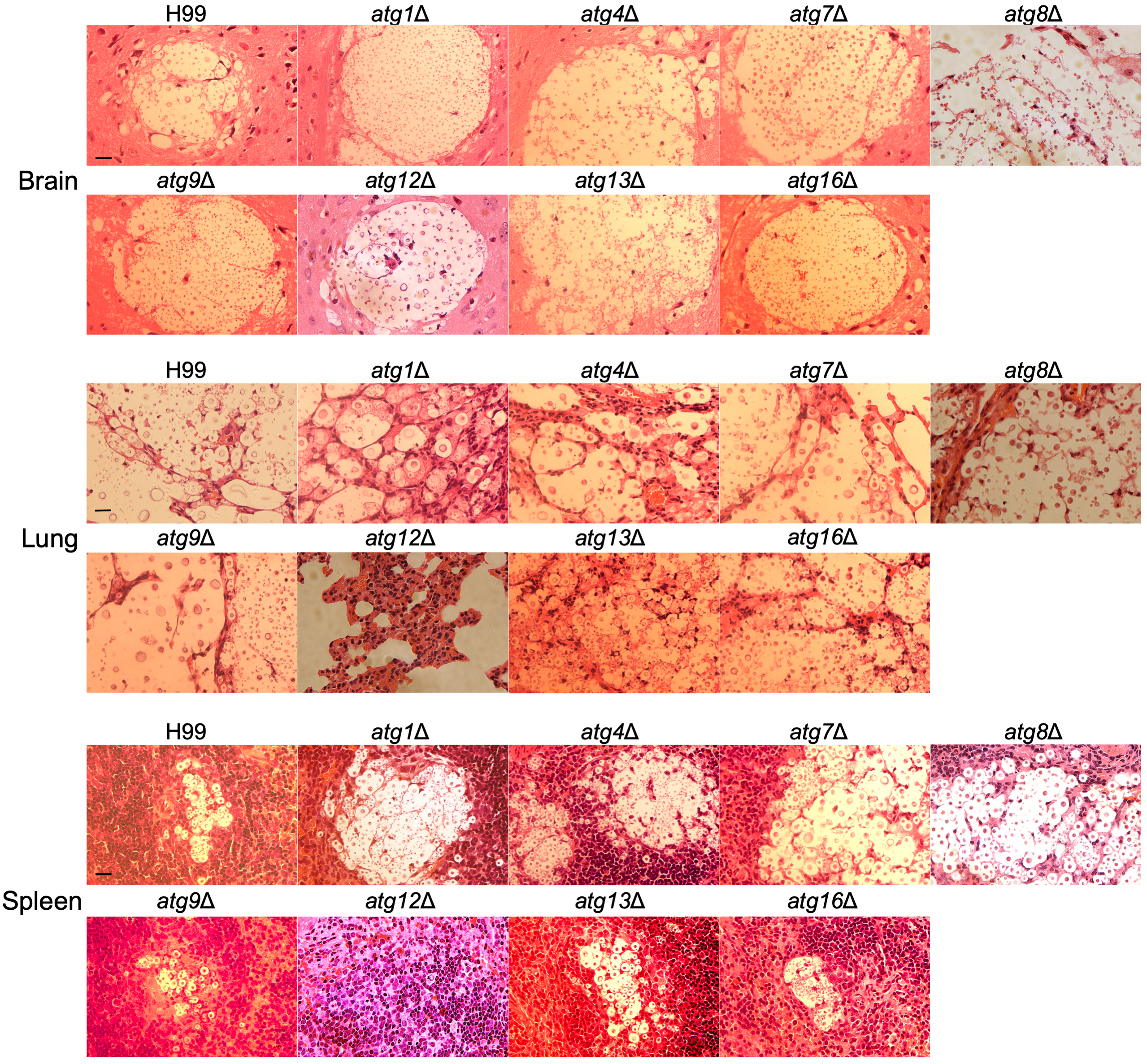

Supplement: Supplementary file 9 [file Image_5.TIF]

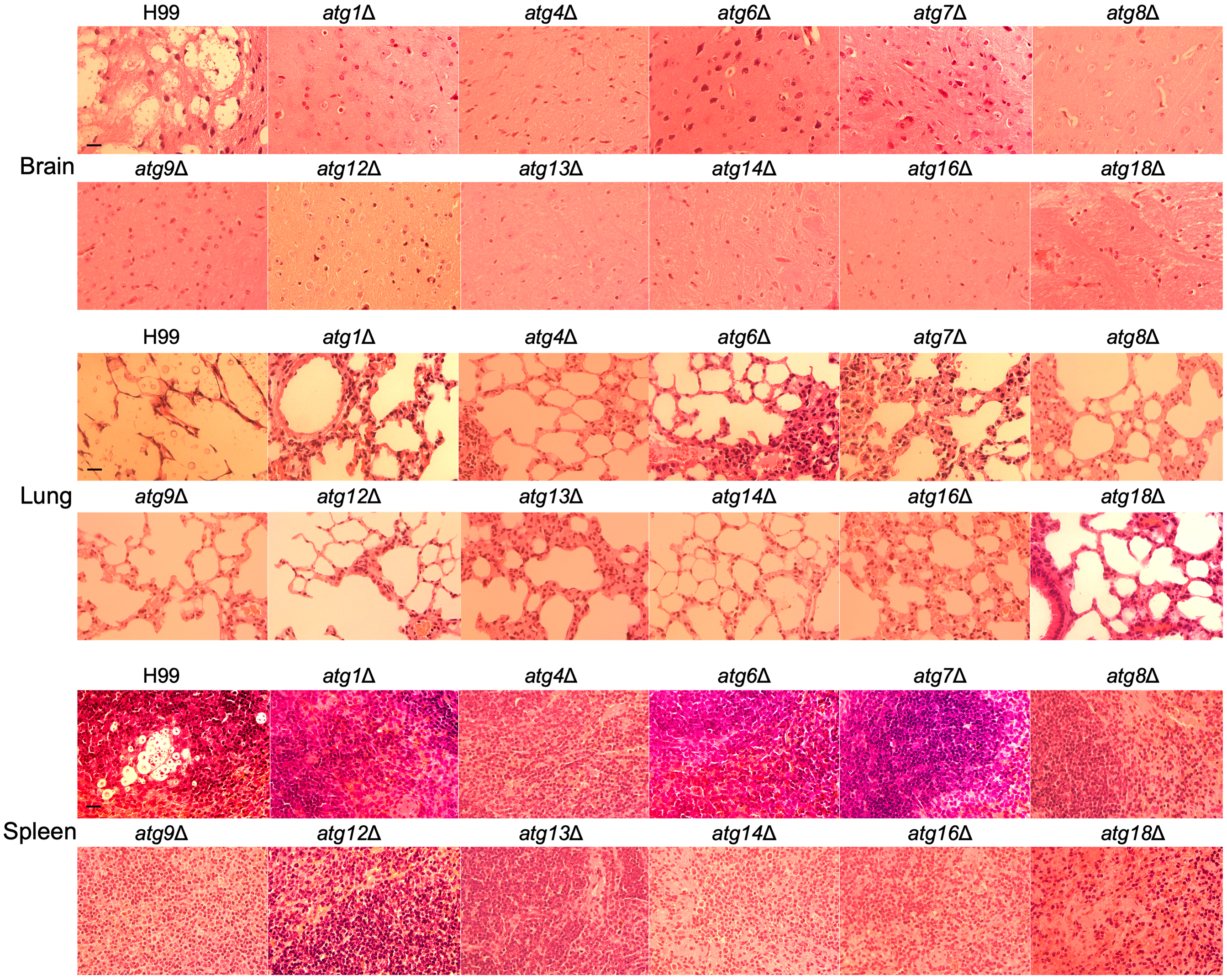

Supplement: Supplementary file 10 [file Image_6.TIF]
